# Natural Medicines for the Treatment of Epilepsy: Bioactive Components, Pharmacology and Mechanism

**DOI:** 10.3389/fphar.2021.604040

**Published:** 2021-03-04

**Authors:** Li-Ying He, Mei-Bian Hu, Ruo-Lan Li, Rong Zhao, Lin-Hong Fan, Lin He, Feng Lu, Xun Ye, Yong-liang Huang, Chun-Jie Wu

**Affiliations:** ^1^ College of Pharmacy, Chengdu University of Traditional Chinese Medicine, Chengdu, China; ^2^ Institute of Pharmaceutical and Food engineering, Shanxi University of Chinese Medicine, Jinzhong, China; ^3^ Hospital of Chengdu University of Traditional Chinese Medicine, Chengdu, China

**Keywords:** natural herbal medicines, epilepsy, bioactive components, mechanisms, therapy

## Abstract

Epilepsy is a chronic disease that can cause temporary brain dysfunction as a result of sudden abnormal discharge of the brain neurons. The seizure mechanism of epilepsy is closely related to the neurotransmitter imbalance, synaptic recombination, and glial cell proliferation. In addition, epileptic seizures can lead to mitochondrial damage, oxidative stress, and the disorder of sugar degradation. Although the mechanism of epilepsy research has reached up to the genetic level, the presently available treatment and recovery records of epilepsy does not seem promising. Recently, natural medicines have attracted more researches owing to their low toxicity and side-effects as well as the excellent efficacy, especially in chronic diseases. In this study, the antiepileptic mechanism of the bioactive components of natural drugs was reviewed so as to provide a reference for the development of potential antiepileptic drugs. Based on the different treatment mechanisms of natural drugs considered in this review, it is possible to select drugs clinically. Improving the accuracy of medication and the cure rate is expected to compensate for the shortage of the conventional epilepsy treatment drugs.

## Introduction

Epilepsy, which is also commonly known as “goatopathy,” was first recognized in 1997, since when the global campaign against epilepsy (GCAE) has been working on the strategy of “improving access, treatment, services, and prevention of epilepsy worldwide” (Saxena and Li, 2017). As per the World Health Organization data on epilepsy for 2006–2015, the number of people with epilepsy continues to remain high. Epilepsy is a common, severe, chronic neurological disease that affects >70 million people across the world. In fact, it affects individuals irrespective of their ages, gender, ethnic background, or the geographic location (Khan et al., 2020).

The known causes of epilepsy has been reclassified as hereditary, structural, infectious, immunological, metabolic, or unknown (Singh and Trevick, 2016). Increasing attention is being paid to the treatment of epilepsy, and the combination of Chinese and western medicine treatment may be more favored (Li, 2012). On one hand, Western medicine treatment for epilepsy can be mainly categorized as etiological treatment, drug treatment, or surgical treatment (Fu and Qu, 2019), example, levetiracetam and phenytoin sodium carbamazepine. On the other hand, natural drugs have been reported to play an important role in the clinical treatment of epilepsy (Piazzi and Berio, 2015). The effect of natural drugs on epilepsy treatment through different mechanisms has been reported in many articles, and the improvement effect is better.

Presently, the conventional drugs that are commonly used for the treatment of epilepsy include carbamazepine, valproate sodium, phenobarbital sodium, phenytoin sodium, and prelampone, among others (Fu and Qu, 2019). These drugs also regulate excitatory and inhibitory discharge in the brain and indirectly regulate the excitatory and inhibitory discharge by regulating the ion concentration. Among these, phenytoin sodium has an outstanding curative effect; however, after treatment, adverse reactions such as anemia after reproduction and acute cerebellar ataxia may occur. Carbamazepine treatment is likely to cause rashes, neurotoxic side effects, diplopia, dermatomyositis, blood and respiratory system damage, and other different types of adverse syndromes. In the recent years, new drugs have been proposed to treat epilepsy, including topiramate, lamotrigine, levetiracetam, and gabapentin. When compared with the conventional drugs, the advantages of these natural drugs include a broad spectrum of antiepileptic, involving less adverse reactions, higher safety, and lesser drug interaction. However, for some refractory epilepsy and epilepsy patients with other comorbidities, the use of these drugs obviously cannot meet their needs. Natural medicines retain the natural and biological activities of their constituents (Guo et al., 2015). Natural drugs have limited or no toxic side-effects. In addition, animals do not possess the advantages of drug resistance, hence the use of natural drugs in animals generally leave no drug residue and causes no health hazards. When compared with the conventional medicine used for the treatment of epilepsy, the composition of natural drugs is complex. Although the use of a single drug may not produce outstanding cure rate, it induces slight toxic side-effects, which can reduce a patient's level of discomfort. The combination of natural and conventional medicines may not only reduce the resultant adverse reactions but also improve the overall comprehensive efficacy (Yuan et al., 2019). Therefore, the present study reviewed the active components of natural drugs and the conventional antiepileptic drugs.

The use of Western medicine alone to treat epilepsy has been reported to induce more adverse reactions. For instance, the use of phenytoin sodium alone can cause gastrointestinal irritation, and its long-term use can cause gingival hyperplasia, nervous system dysfunction, and hematopoietic system disorders. The use of carbamazepine alone may induce dizziness, nausea, vomiting, and ataxia as well as occasional aplastic anemia and granulocytopenia. Valproate alone can cause nausea, vomiting, lethargy, tremor, hair loss, and hepatotoxicity. The use of an antiepileptic alone may induce anorexia, nausea, dizziness, and drowsiness.

The present paper reviewed the effects of active components of natural drugs on epilepsy, including flavonoids, alkaloids, glycosides, coumarins, and terpenoids. Among the monomer components, flavonoids, alkaloids, and terpenoids demonstrated significant activity against epilepsy. We have summarized the methods for the prevention and treatment of epilepsy by balancing excitatory and inhibitory neurotransmitters and inhibiting neuroinflammation, oxidative stress, and mitochondrial dysfunction. In addition, we innovatively summarized the combined methods of natural drug monomer compounds and natural drug compound as well as conventional antiepileptic drugs, expecting to bring hope to epileptic patients and provide them with a credible reference for improving the epilepsy cure rate.

## The Pathogenesis of Epilepsy

One of the main causes of epileptic seizures is believed to be the abnormal activity of cortical neurons, and the abnormal discharge of these neurons has mostly been related to the loss of specific subarea inhibitory and excitatory neurons, neurotransmitter transmission and imbalance, synaptic recombination, axonal germination, as well as the change in the glial cell functioning and structure. Glial cells and axons in the white matter content may play a secondary role in this situation (Xue, 2005; Zhu et al., 2014). Recurrent seizures can lead to abnormal synaptic protein expression, synaptic remodeling, and abnormal neuronal network formation, which is one of the pathophysiological mechanisms of refractory epilepsy (Yang et al., 2017). In addition, with the development of molecular biology, the study of epilepsy mechanism has shifted from phenotype to genotype, with dozens of genes or candidate genes found. The occurrence of epilepsy can be attributed to primary genetic abnormality or secondary definite structure or metabolic disorder (Depondt, 2006; Thijs et al., 2019). Genealogy and genetic analysis have indicated that epilepsy can be inherited in one or more genes, dominant or recessive, or even concomitantly. Therefore, innate genetic factors and acquired environmental factors can lead to the occurrence and development of epilepsy.

### Synapses and Receptors

GABA is a major inhibitory neurotransmitter in the cerebral cortex that maintains the inhibitory tension to balance nerve excitation (Hirose, 2014). If this balance is disturbed, seizures follow. The enzyme glutamic acid decarboxylase (GAD) can promote the synthesis of neuronal GABA from glutamic acid, which is encoded by two different genes, *GAD2* and *GAD1* (Obata, 2013). GAD1 plays the major role for GABA production in the embryonic brain, whereas the contribution of GAD2 begins to increase after birth. GAT-1 and GAT-3 are GABA-transporters (GATs), and high level of GAT content has been associated with seizures. GABA receptors can be categorized into three types based on their different pharmacological characteristics as GABA-A, GABA-B, and GABA-C receptors (Pham et al., 2016). GABA-type A receptor (GABA-A R) has been found to be the major genetic target of heritable human epilepsy (Chen et al., 2017). GABA-A induces epilepsy mainly in the following ways: through controlling the chloride ion flow or by impairing GABAergic inhibitory input that lead to synchronous excitatory activity in the neuronal population and, ultimately, seizures (Beenhakker and Huguenard, 2009). GABAB, which increases potassium conductance, reduces the Ca^2+^ entry and inhibits the release of other presynaptic transmitters. Presently, reduced or abnormal GABA function has been detected in both genetic and acquired animal models of epilepsy and in the human epileptic brain tissues (Treiman, 2001).

Glutamate (Glu) acts on various membrane receptors, which form cation-permeable ion channel receptors. It can be categorized into three families: alpha-amino-3-hydroxy-5-methyl-4-isoxazole-propionate (AMPA) receptors (AMPARs), kainate receptors (KARs), and NMDA receptors (NMDARs) (Paoletti et al., 2013). The high levels of Glu causes nerve damage or death, mainly due to increased NMDAR activity and Ca^2+^ influx through the NMDAR channels. Excessive NMDAR activity may also form the basis for epileptic seizures, which are characterized by neuronal hyperexcitability or sensitivity. In addition, nicotinic acetylcholinergic receptors (nAChRs) in the vertebrates are pentammer ligands-gated ion channels assembled from homologous subunits (Adams et al., 2012). Central nAChRs can influence the onset of epilepsy through the regulation of the release of other neurotransmitters, such as glutamate, GABA, dopamine, and norepinephrine (Sinkus et al., 2015). Several past studies have suggested that nNOS can facilitate seizure generation during SE. The mechanism involved in this event is that NO reduces the blood-brain barrier opening after trauma by improving the vascular permeability (Gangar and Bhatt, 2020). The 5-HT receptor-related changes have also been reported in the study of epilepsy mechanisms (Zhao et al., 2018).

### Ion Channels and Epilepsy

Mutations in the sodium channels are responsible for the development of genetic epilepsy syndromes with a wide range of severity. Mutations in the NaV1.1 channels have severely impaired sodium currents and the action potential firing in the hippocampal GABAergic inhibitory neurons, which can cause hyperexcitability, contributing to seizures (William et al., 2010). SCN1A gene, which encodes NaV1.1 subunit expressed in inhibitory GABA neurons, has also been implicated in the mutations of *SCN1A* in epilepsy patients (Duflocq et al., 2008). In addition, SCN1B, SCN2A, and SCN8A mutations have been found to be associated with epilepsy (Tang and Mei, 2016). Potassium channels are the most diverse group of ion channels and they play an important role in countless cellular processes, for example, in regulating the potassium outflow, current and action potential, and neurotransmitter release (Contet et al., 2016). The K^+^ channels control the resting membrane potential and enable rapid repolarization of the action potential by producing outward K^+^ currents, which limits neuronal excitability. Among the numerous genes that encode potassium channels, mutations in KCNMA1 were first reported in large families with autosomal-dominant totipotent epilepsy and parasympathetic dysmotility. Subsequently, mutations in KCNQ2, KCNT1, and KCNQ3 were reported in familial neonatal epilepsy (Tang et al., 2016).

A large amount of Ca^2+^ influx not only causes excitatory amino acid poisoning but also increases the concentration of Ca^2+^ in the cells, thereby inducing neuronal damage. The plasmids become overcharged with negative Ca^2+^ influx for a long time. The Ca^2+^ imbalance and the malfunctioning of mitochondria form a vicious cyclemake the brain organization of ATP production insufficient, the release of mPTP leads to fine cytosolic edema, and results in intracellular Ca^2+^ overload of the nerve cells, which eventually causes nerve cell death. In addition, the overload leads to excessive production of NO in the regulatory neurons, which can be combined with superoxygenated substances to produce ONOO- in the nervous cells; this event is highly toxic to the white matter, membrane lipids, and DNA as well as leads to oxidative stress. Therefore, the increase in Ca^2+^ concentration affects the occurrence of epilepsy from different aspects. Currently, numerous experimental data suggest that CACNA1A mutation plays a significant role in human epilepsy (Alexander et al., 2016). Moreover, the CLCN2 channel plays a critical role near GABA-A receptors at the GABAergic inhibitory synapses (Agostino et al., 2004). CLCN2 mutations in multiphenotypic families (Martin et al., 2009) and in primary systemic epilepsy have also been identified. The mechanism of epilepsy induced by the imbalance of the CLC channels or gene mutation may be related to its regulation of excitability of the cell membrane and the transport functions of electrolytes, water, and nutrients (Wei et al., 2017). Highly polarized activated cyclic nucleotide gating (HCN) channels encoded by 4 genes (HCN1-4) have been reported to undergo transcriptional changes in patients with epilepsy, with the possible mechanism of influencing excitability in patients with epilepsy (Difrancesco and Difrancesco, 2015).

### Immune System

Impaired immune function and inflammatory response are both the cause of occurrence and development of epilepsy as well as the result of partial epilepsy. Past studies have demonstrated that CD3, CD4, and CD4^+^/CD8^+^ count of helper T-cells decrease and the CD8^+^ value of inhibitory T-cells increase significantly in the peripheral blood of epileptic patients (DiFrancesco and DiFrancesco, 2015; Arreola et al., 2017). In addition, several past scholars have studied the changes in the values of IgG, IgA, IgM, IgG1, IgG2, IgG3, and IgE in epileptic patients (Callenbach et al., 2003; Godhwani and Bahna, 2016). A weakened immune system often acts as an accomplice in the onset of epilepsy, along with other trigger factors (Matin et al., 2015). Moreover, cytokines involved in the regulatory effects of the immune system have been found to be involved in epilepsy in patients with partially overexpressed states. A major portion of this process is the inflammatory response, and changes in the inflammatory factors to a certain extent indicate that the occurrence of epilepsy can also induce a certain inflammatory response. For instance, IL-1β, IL-6, IL-10, IL-2, IL-17 (Kumar et al., 2019), IL-4, and TNF-α were abnormally expressed in patients, whose elevated levels can lead to neuronal degeneration and induce epilepsy (Ravizza et al., 2006). Another mechanism by which IL-1 participates in epilepsy is through the upregulation of NMDA receptors on postsynaptic cells through the activation of GluN2B subunits of NMDA receptors (Ravizza et al., 2006). TNF-α increases the number of Glu receptors and induces the ingestion of GABA, which in turn reduces the inhibitory drive and induces neuronal excitation, which leads to the development of epilepsy (Liu et al., 2015).

### Glioma-Associated Epilepsy

Glial cells regulate excitatory and inflammatory responses that affect the occurrence of epilepsy (Devinsky et al., 2013). Astrogliosis is a common pathological hallmark of idiopathic and acquired forms of epilepsy. l-glutamic acid, d-serine, GABA, and kynurenic acid released from astrocytes are mostly involved in the epileptic process (Yamamura et al., 2013). In addition, GS is a cytoplasmic enzyme present in astrocytes that regulates the Glu acid levels. In a past study, the GS levels were significantly reduced in the hippocampus and the amygdala of TLE patients, which suggests that this enzyme is associated with the occurrence of epilepsy (Devinsky et al., 2013). Microglia activation not only increases the levels of brain inflammatory factors and TNF-α but also enhances the activities of induced nitric oxide synthase (iNOS) and cyclocycox-2 (COX-2), which can enhance the induction of epilepsy induced by neurogenesis (Akin et al., 2011; Yuan and Liu, 2020). The activated astrocytes induce the release of inflammatory factors such as IL-1β. Therefore, glial cells are not only involved in the imbalance of neurotransmitters in the process of epilepsy but also in the process of inflammation.

### Mitochondrial Dysfunction and Oxidative Stress

Mitochondrial oxidative stress and dysfunction may trigger epileptic seizures arising from mitochondrial DNA (mtDNA) or nuclear DNA mutations and temporal lobe epilepsy (Rowley and Patel, 2013). Myoclonic epilepsy has been shown to be associated with mtDNA mutations. Two such targets of oxidation related to episodes of epilepsy are the glial glutamate transporters GLT-1 and GLAST (Liang et al., 2012). In addition, oxidative stress and mitochondrial dysfunction result from prolonged duration of seizure. The depolarization pattern during intense epileptic activity of neurons in response to external stimuli leads to mitochondrial depolarization and mitochondrial Ca^2+^ accumulation, which in turn induces mitochondrial apoptotic pathways or oxidative stress that accelerate energy failure and mitochondrial superoxide production (Kudin et al., 2002). Superoxide is a moderately active free radical and its production leads to the formation of more active ROS, which lead to lipid peroxidation and subsequent membrane destruction that are reflected in the increased content of MDA as a product of lipid peroxidation. This event thus promotes the intrinsic pathway toward triggering of cell apoptosis and death. The content of SOD, CAT, and glutathione peroxidase in the mitochondria also changes in this situation. The degradation pathways of superoxide and hydrogen peroxide (H_2_O_2_) involved in SOD and CAT were also affected with the change in the upstream products. The time-dependent generation of H_2_O_2_ in the hippocampal mitochondria as well as the frequency of mtDNA damage also increases in epileptic patients (Smith and Patel, 2017).

### Glycogen Degradation

Glycogen is involved in the neurotransmission of glutamate as well as in the degradation of glycogen to promote glutamate transport in the astrocytes. Furthermore, glycogen in astrocytes can be used to synthesize glutamine, which is a precursor of glutamate (Bark et al., 2018). In addition, glycogen is involved in promoting the removal of K^+^ from the extracellular space, and excessive neuronal activity has been associated with K^+^ efflux (Walls et al., 2009). Moreover, decreased glycogen degradation is believed to be associated with epileptic seizures.

### Glucocorticoids

Glucocorticoids have been reported to be involved in the regulation of various activities of the nervous system through the GR (Kanner, 2009). Glucocorticoids have also been reported to affect people with epilepsy. Some of the reported results demonstrate that the level of glucocorticoids increases in epilepsy models. However, only a few studies have been reported in this field, although this conclusion needs further verification. The possible pathogenesis of epilepsy is displayed in Table 1.

**TABLE 1:** Possible mechanisms involved in epilepsy.

Component	Specific factors	References
Neurotransmitters	Imbalance of Glu and GABA	Hirose (2014)
Synapses and Receptor	GABA-A, NMDA, 5-HT, AMPA receptor, acetylcholine receptor Enzyme, modulator, transporter, axonal burst bud	Hirose (2014); Paoletti et al. (2013); Thijs et al. (2019)
Ion channels	Sodium channel; Potassium channel; HCN channel; Calcium channel; Chloride channel	Alexander et al. (2016); Contet et al. (2016); Duflocq et al. (2008); Tang et al. (2016)
Inflammatory cytokines	IL-1β, IL-2, IL-4, IL-6, IL-10, TNF-α, cyclooxygenase-2, Platelet-activating factor, Prostaglandin E2, Adhesion molecules, MMP-9; TLR-1, -2, -3; Chemokines were increased	Godhwani and Bahna (2016); Kumar et al. (2019)
Immune system	Both cellular and humoral immunity are affected. Increasing IgA, IgG, CD8, CD54; Decreasing CD3, CD4, CD4/CD8	Ravizza et al. (2006); Roseti et al. (2015); Liu et al. (2015)
Glial cell	Astrocytes and microglia proliferated and the ability of astrocytes to absorb potassium ions decreased	Bark et al. (2018)
Oxidative stress and apoptosis	ROS was increased, the ratio of Bcl-2/Bax was decreased, and the expression levels of apoptotic proteins cytochrome C and Caspase-3 were significantly increased	Liang et al. (2012); Kudin et al. (2002)
Mitochondrial dysfunction	Increasing Ca^2+^ and ATP consumption	Rowley and Patel (2013); Liang et al. (2012); Kudin et al. (2002); Smith and Patel (2017)
Genetic factors	SCN1A, SCN2A, SCN8A and other mutations	Pal et al. (2010)
Glycogen metabolism	Decreased glycogen degradation, abnormal expression of glucocorticoid	Walls et al. (2009)

MMP-9, Matrix metalloproteinase-9; TLR, Toll-like receptors.

## Pharmacological Effects of Natural Medicines for the Management of Epilepsy

Despite the increasing number of researches on natural medicine, the ingredients of natural medicine remain complex, such as alkaloids, flavonoids, saccharides, glycosides, quinones, coumarins, lignans, terpenes, volatile oils, saponins, and cardiac glycosides (Kim, 2016). Several ingredients have been reported to possess antiepileptic activity, with the main therapeutic mechanisms including regulating synapse and receptor pathways (i.e., GABA, Glu, NMDAR, and 5-HT), ion channels (i.e., Ca^2+^, K^+^, and Na^+^), immune system (i.e., CD3, CD4, IgG, IgA, TNF-α, IL-1β, IL-2, IL-4, IL-6, and IL-10), glial cells (i.e., lial cell proliferation and potassium uptake ability) and mitochondrial dysfunction and oxidative stress (i.e., oxidation markers, accumulation of Ca^2+^, cell death, and apoptosis). Presently, we can reclassify these ingredients in accordance to the difference in the mechanisms of action.

## Natural Medicines Improves Epilepsy by Regulating Synapses and Receptors

Flavonoids share similar structures to benzodiazepines (Nilsson and Sterner, 2011), and play an anti-epileptic role through the regulation of the GABAA-Cl-channel complex (Xiang et al., 2014). Several flavonoids that can be used to treat epilepsy through different receptor signaling pathways are known. Tanshinone IIA is a hydrophobic ketone extracted from *Salvia miltiorrhiza*. Past studies have shown that the reduced c-fos expression in the brains of PTZ-exposed zebrafish larvae plays a therapeutic role in epilepsy through the activation of the GABA signaling pathway (Buenafe et al., 2013). Curcumin can improve depressive behavior and cognitive functioning through inhibition of acetylcholinesterase and by mediating monoaminergic regulation, which significantly reduces the number and degree of seizures by via inhibition of the activation of mechanistic target of the rapamycin complex 1 (mTORC1) (Kiasalari et al., 2013). Amentoflavone can improve the activity of hippocampal acetylcholinesterase as well as the learning and memory functions of rats (Yuan, 2016). However, amentoflavone has no regulating effect on the GABAR channel current in insular neurons induced by GABA. Luteolin is one of the main isolates of resveratrol, which is a natural flavonoid (Shen et al., 2016). Past studies have demonstrated that luteolin can increase the seizure threshold, and the mechanism may be to enhance the activation of GABAA receptors, thereby promoting the opening of GABA-mediated chlorine channels (Tambe et al., 2016; Tambe et al., 2017). (-)-Epigallocatechin-3-Gallate (EGCG) plays a therapeutic role in lithium-pilocarpine-induced epilepsy through the inhibition of the Toll-like receptor 4 (TLR4)/nuclear factor-κB (NF-κB) signaling pathway (Qu et al., 2019). Nobiletin significantly upregulated the expression of GAD65 and GABAA. Bupleuronin inhibited the current generated by NMDA receptor activation (Yang et al., 2018).

Glycosides have been reported to exert a therapeutic effect on epilepsy. Paeoniflorin (PF) plays an antiepileptic role through the inhibition of glutene-induced Ca^2+^ influx, activation of the metabotropic Glu receptor 5 (mGluR5), membrane depolarization, and neuronal death induced by Glu (Hino et al., 2012). Gastrodin mainly involves antioxidants and regulates the release of neurotransmitters. It has been reported to decrease GABA-T, GAD65, and GAD67 (Yuan et al., 2019). Sclerosylglucoside usulate (UASG) significantly prolonged the incubation period and reduced the seizure duration in animal models of INH-induced epilepsy by increasing GABA release (Kazmi et al., 2012).

Recently, the application of terpenoids in epilepsy has attracted much attention. (+)-Dehydrofukinone (DHF), an active ingredient in *Acorus tatarinowii*, is believed to possess anticonvulsant properties and may act as a potential antiepileptic drug through the induction of sedation and anesthesia via modulation of GABAA receptors (Garlet et al., 2017). In addition, 1-nitro-2-phenylethane is an active component that is isolated from volatile oil of *Aniba canelilla*. In a past study, mice injected with 1-nitro-2-phenylethane flumazine showed prolonged sleep pattern, and this hypnotic effect was possibly due to the upregulation of GABA in the central nervous system (Oyemitan et al., 2013). Tetrahydrocannabinol (THC) has been the primary focus of cannabis research until date. Delta9-THC (∆9-THC) exerts antioxidant effects in α-amino-3-hydroxy-5-methyl-4-isoxazolepropionic acid models and NMDA-mediated cytotoxicity models. Moreover, ∆9-THC also desensitizes and transiently activates the transient receptor potential (TRP) channels TRPA1, TRPV1, and TRPV2 (Bahr et al., 2019). Borneol can easily cross the blood-brain barrier and exert a certain GABA regulating effect (Xiang et al., 2014). Isopulegol exhibited anticonvulsive effects through the positive modulation of benzodiazepine-sensitive GABAA receptors and antioxidant properties.

Alkaloids have been reported to regulate different receptors. Rhynchophylline (RIN) is an alkaloid isolated from *Uncaria rhynchophylla* (Ho et al., 2014). On one hand, RIN decreases neuronal hyperexcitability by inhibiting NMDA receptor current, and further decreases the expression of N-methyl-d-aspartate receptor 2B (NR2B) protein induced by pilocarpin (Shao et al., 2016). On the other hand, RIN inhibits the synaptic transmission. Sanjoinine A is an alkaloid active ingredient isolated from *Zizyphi Spinosi Semen* (Ma et al., 2008). Sanjoinine A not only blocks NMDA-induced epileptoid electroencephalography changes and reduces cerebellar granulosa cell damage but also inhibits intracellular Ca^2+^ influx in NMDA-induced models. Tetrahydropalmatine (THP) is an alkaloid component isolated from *Rhizoma corydalis*. When THP was injected into the epileptic model, dopamine secretion was reduced in parallel with the enhancement in GABA receptor function and cholinergic receptor function and the kindling process was inhibited (Lin et al., 2002). Tetramethylpyrazine (TMP) is the main bioactive alkaloid in *Chuanxiong Rhizoma* (*Conioselinum anthriscoides* ‘*Chuanxiong*’). The therapeutic effect of TMP on epilepsy may be attributed to its inhibitory excitatory synaptic transmission (Jin et al., 2019). Berberine acts as an anticonvulsant through the regulation of the neurotransmitter system. It can reduce the hyperexcitatory movement and abnormal movement trajectory of the larvae in a concentration-dependent manner, which slows down excessive photosensitive epileptiform swimming and helps restore the STX1B expression level to balance (Zheng et al., 2018).

Coumarin is the general name of o-hydroxycinnamate lactones, which possess an effect of anticoagulant, photosensitive, antibacterial, cytotoxic, antioxidant, and neuroprotective activities. The anticonvulsant effects of coumarin have been found to be related to the regulation of GABA receptors. For example, esculetin (6,7-dihydroxycoumarin) has been reported in animal models to induce an antiepileptic effect through the regulation of GABA neurons. However, this effect needs to be tested further to comprehend how such high concentrations of the substance cross the blood-brain barrier into the brain. The mechanism of coumarins in epilepsy is hence not considered may be not entirely through the GABAA receptors (Xiang et al., 2014).

**FIGURE 1:**
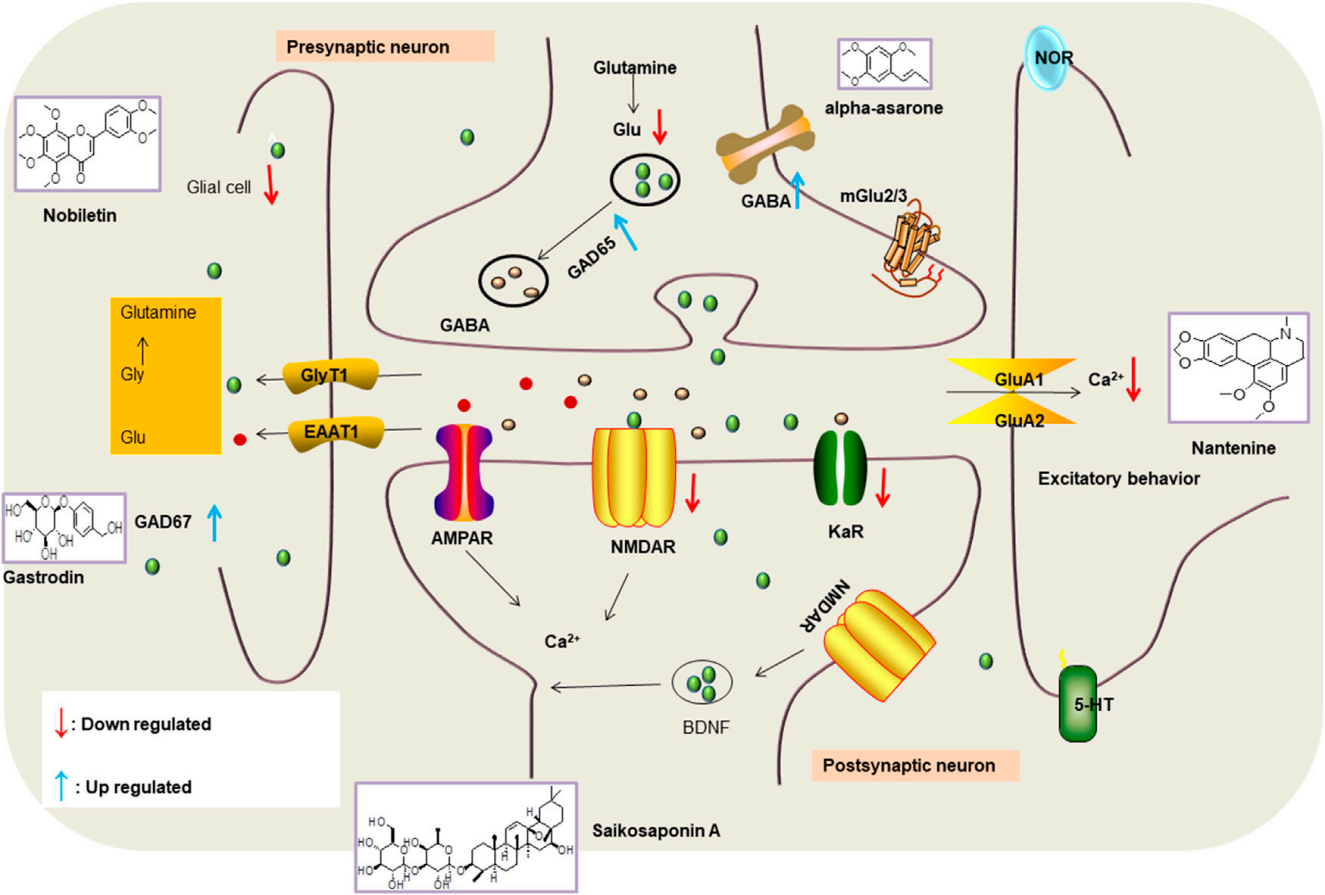
Effects of natural drugs on receptor pathway. α-amino-3-hydroxy-5-methyl-4-isoxazolepropionic acid receptor (AMPAR), brain-derived neurotrophic factor (BDNF), excitatory amino acid transporter (EAAT), -aminobutyric acid (GABA), glutamate decarboxylase (GAD), glutamate (Glu), glycine (Gly), N-methyl-D-aspartate receptor (NMDAR).

Various anti-epileptic mechanisms of α-asarum have been reported, which may be mainly related to neurotransmitters and apoptotic factors. α-Asarum decreased the activity of GABA-T, while the expression of GAD 67 and GABAA receptors were increased (Miao et al., 2011). In addition, the α-asarum level can obviously reduce the expression of NMDA receptor-l level in the hippocampus CA1 and CA3 areas, thereby inhibiting the activity of NMDARl and excitatory neurotoxicity (Liu et al., 2013). We noted that flavonoids, terpenoids, and alkaloids play an important role in the treatment of epilepsy through the receptor pathways. The main receptors affected are the Glu receptor pathway and the GABA receptor pathway. More natural compounds that play antiepileptic roles through the regulation of receptors and synaptic pathways are displayed in Table 2. The effects of natural drugs on the receptor pathways are depicted in Figure 1.

**TABLE 2:** Natural drugs used to treat epilepsy by regulating neurotransmitters and synaptic function.

Natural drugs	Compounds	Chemical structure	Animal models	Mechanisms	References
*Salvia miltiorrhiza Bunge* (*Lamiaceae*)	Tanshinone IIA	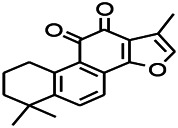	PTZS in rats; 4-APS in mice	Activating GABA signaling pathway; Decreasing MEK activity and Glu, C-fos expressions	Buenafe et al. (2013); Tan et al. (2014)
*Curcuma longa L.* (“*turmeric*,” *Zingiberaceae*)	Curcumin	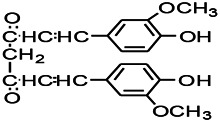	PTZS and KAS in mice	Inhibiting acetylcholinesterase and mediating monoaminergic regulation	Kiasalari et al. (2013)
*Passiflora coerulea L. var. Hort.*	Chrysin	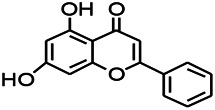	PTZS in rats	Myorelaxant action agonizing the benzodiazepine receptor	Xiang et al. (2014)
*Matricaria chamomilla L.* (*Chamomile*)	Apigenin	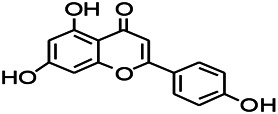	PTXS in mice	Curbing benzodiazepine agonist	Xiang et al. (2014)
*Scutellaria baicalensis Georgi.* (*Lamiaceae*)	Wogonin	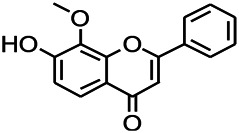	Mice	Enhancing expression of GABAA receptors	Diniz et al. (2015)
*Bupleurum chinense* (*Umbelliferae*)	Quercetin	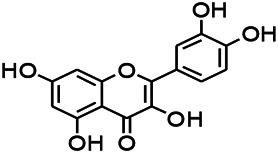	KAS	Influencing ionotropic GABA receptors	Xiang et al. (2014)
*Plantago asiatica L* (*Plantaginaceae*)	Hispidulin	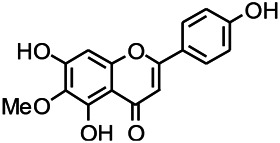	Rat	Decreaseing Glu	Diniz et al. (2015)
*Green tea* (*Camellia sinensis (L.) Kuntze*)	EGCG	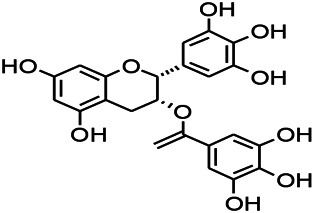	L&PS in rats	Increasing impression of GABA	Qu et al. (2019)
*Withania somnifera (L.) Dunal* (*Solanaceae*)	Withanolide A	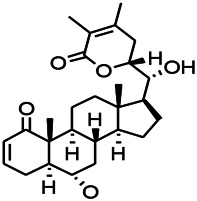	PTZS in rats	Recovering distorted NMDA receptor solidity; Regulating AMPA receptor function	Xiang et al. (2014)
*Maclura tinctoria (Moraceae)*	Morin	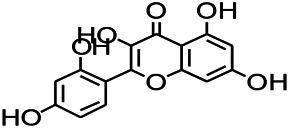	PTZS in mouse	Modulating the concentrations of GABA	Lee et al. (2018)
*Radix astragali (Astragalus species)*	Baicalin	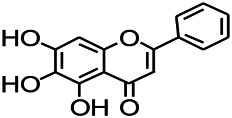	PTZS in rats	Increasing impression of GABA	Diniz et al. (2015)
*Citrus reticulata Blanco* (*Rutaceae*)	Nobiletin	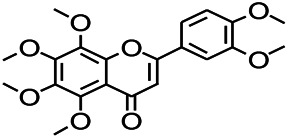	PTZS in mice	Modulating expression of GABAA and GAD65; Recrovering Glu and GABA balance	Yang et al. (2018)
*Smoke tree* (*Cotinus coggygria*)	Fisetin	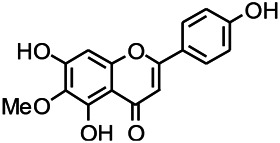	Iron-induced experimental model in rats	Increasing GABA level in brain	Diniz et al. (2015)
*Gastrodia elata Blume* (*Orchidaceae*)	Gastrodin	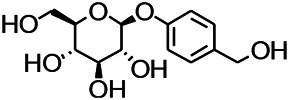	NMDAS in rat	Decreasing GABA-T, Glu, Increasing GAD65, GAD67	Liu et al. (2018)
*Radix bupleuri* (*Bupleurum L.*)	Saikosaponin A	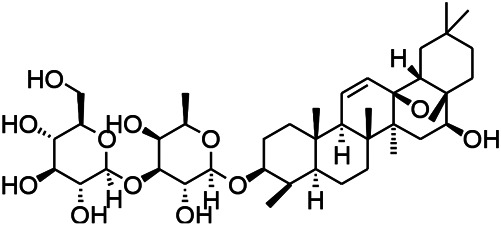	L&PS in rats	Decreasing protein of p-gp, NMDAR	Xiang et al. (2014); Xie et al. (2013)
*Lantana camara L.* (*Verbenaceae*)	Ursolic acid Stearoyl glucoside	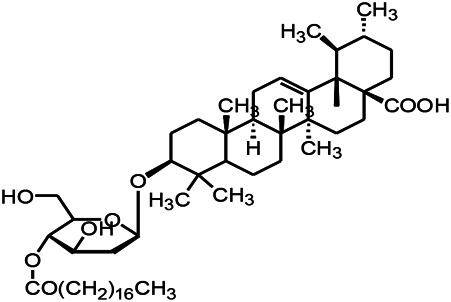	MES in mice	Increasing the GABA level in central nervous system	Kazmi et al. (2012)
*Curcuma longa L. (Zingiberaceae)*	Curzerene	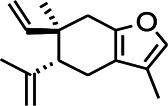	PTZS in mice	Effecting GABAergic and opioid systems	Abbasi et al. (2017)
*Rhododendron tomentosum (Ledum palustre)*	P-Cymene	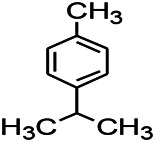	MES in mice	Mediating an increase in GABAergic response	Abbasi et al. (2017)
*Matricaria chamomilla L. (Lauraceae)*	(+)-Dehydrofukinone	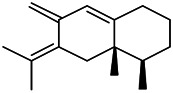	GABAA RM in mice	Facilitating GABAergic neuronal	Garlet et al. (2017)
*Dennettia tripetala Baker f (Pepperfruit)*	1-nitro-2-phenylethane	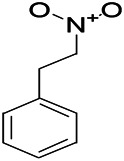	PTZS in mice	Associated with GABA neurons	Oyemitan et al. (2013)
*Nigella sativa (N. sativa) L. (Ranunculaceae)*	Alpha-Pinene	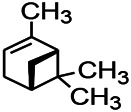	PTZS in mice	Mediating GABAergic response	Bahr et al. (2019)
*Thymus vulgaris L. (Lamiaceae)*	Terpinen-4-ol	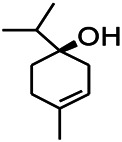	PTZS in mice	Regulating GABAergic neurotransmission	Bahr et al. (2019)
*Various medicinal plants*	Phytol	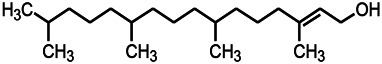	PTZS in mice	Interacting with GABAA receptor	Xiang et al. (2014)
*Ginkgo biloba L. (Ginkgoaceae)*	Bilobalide	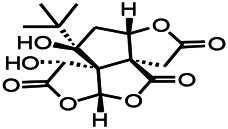	L&PS in rats	Increasing GABA levels by potentiation	Xiang et al. (2014)
*Capsicum annuum L. (Solanaceae)*	Carvacrol	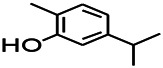	PTZS, MES in mice	Enhancing GABAA BZD receptor	Xiang et al. (2014)
*Saffron (Crocus sativus L.)*	Safranal.	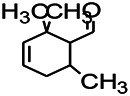	PTZS in rats	Regulating the GABAA benzodiazepine receptor	Xiang et al. (2014)
*Uncaria rhynchophylla (Miq.) Miq. exHavil. (Uncaria Schreber nom. cons.)*	Rhynchophylline isorhynchophylline	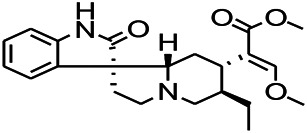 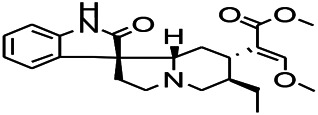	KAS in mice	Decreasing central nervous system synaptic transmission	Shao et al. (2016); Wang and Cai (2018)
*Piper nigrum L. (Piperaceae)*	Piperine	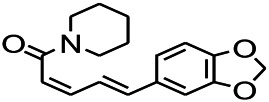	PTZS in both zebrafish and mice	Increasing GABA; Inhibit the TRPV1 receptor	Diniz et al. (2015)
*Aconitum carmichaeli Debx. (Ranunculaceae)*	Aconitine	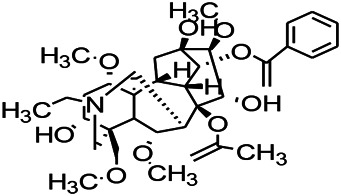	Male Wistar rats	Blocking GABAA mediated	Zhao et al. (2020)
*Aconitum carmichaeli Debx. (Ranunculaceae)*	3-Acetylaconitine	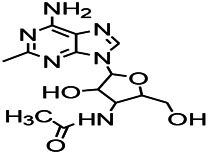	Male Wistar rats	Promoting GABAA activity	Zhao et al. (2020)
*Aconitum carmichaeli Debx. (Ranunculaceae)*	Lappaconitine	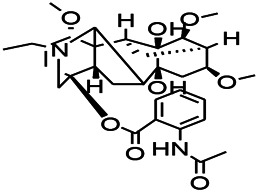	Male Wistar rats	Promoting the release of GABAA	Xiang et al. (2014)
*Aconitum carmichaeli Debx. (Ranunculaceae)*	Montanine	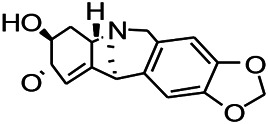	PTZS in rats	Modulating neurotransmitter receptor systems; including GABAA receptors	Xiang et al. (2014)
*Coptis chinensis Franch., C.(Ranunculaceae)*	Berberine	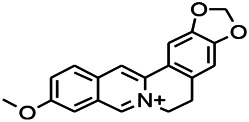	PTZS in Zebrafish	Reducing convulsions and mortality and NMDA	Yang et al. (2018)
*Erythrina mulungu Mart ex Benth (Leguminosae-Papilionaceae)*	Erysotrine	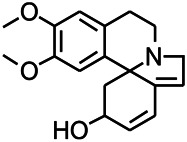	PTZS in miceBCLS in rats	Related to NMDA	Xiang et al. (2014)
*Cortex fraxini (Fraxinus rhynchophylla Hance)*	Esculetin	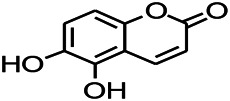	EMS in mice	Probably through the GABAergic neuron	Xiang et al. (2014)
*Acorus tatarinowii Schott (Acorus L. Araceae)*	α-asarone	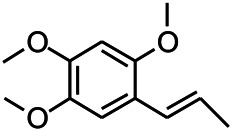	L&PS in rats	Decreasing GABA-T and NMDAR1 mRNA. Increasing GAD65, GAD67	Miao et al. (2011); Yuan and Liu (2020)
*Acorus tatarinowii Schott (Acorus L. Araceae)*	β-asarone	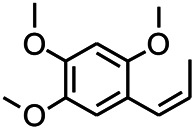	PTZS in rats	Modulating the excitatory transmitter glutamate	Yuan et al. (2019)
*Nandina domestica Thunb (Berberidaceae)*	Amentoflavone	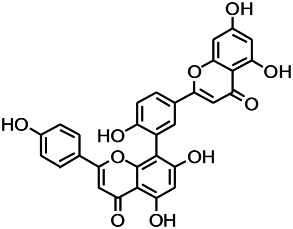	L&PS in rats	Improving the activity of acetylcholinesterase	Yuan (2016)

4-AP induced seizures; EGCG, (-)-Epigallocatechin gallate; BCLS, bicuculline induced seizure; GABAA RM, GABAA Receptor mulation; KAS, Kainic acid (KA)-induced seizures; L&PS, Lithium & pilocarpine induced seizures; MES, Maximal electroshock-induced seizures; NMDAS, NMDA induced seizures; PTZS, PTZ-induced seizures; PTXS, Picrotoxin induced seizures.

## Natural Medicines Improves Epilepsy by Regulating Ion Channels

Tanshinone IIA, a flavonoid compound, activates the potassium channels by increasing the presynaptic Ca^2+^ influx and improves the cognitive function of epileptic rats (Lin et al., 2013; Tan et al., 2014).

Glycosides and saponins can play some roles by regulating the ion channels. For example, ginsenoside Rg3 can inhibit seizure-induced Ca^2+^ influx with spontaneous recurrent epileptiform discharges (SRED) and further attenuate SREDs-induced neuronal death (He et al., 2019). PF is a water-soluble monoterpenoid glycoside extracted and isolated from *Paeonia lactiflora Pall*. The anti-epilepsy mechanism of PF may be involved in inhibiting the increase of intracellular Ca^2+^ influx (Hino et al., 2012).

Terpenoids can also play a role by regulating the ion channels. For example, triptolide (TL) exerts neuroprotective effects in epileptic rats, possibly by increasing the expression of kv1.1 in the hippocampus CA3 (Pan et al., 2012). The specific effect of eugenol on the ionic current has been shown to increase the degree of voltage-gated Na^+^ current inactivation and inhibiting the non-inactivated (Kim, 2016).

The ionic regulation of alkaloids has been reported in several articles. RIN decreased neuronal hyperexcitability by inhibiting continuous sodium current (INaP) and further decreased pilocarpin-induced Nav1.6 (Shao et al., 2016). RIN also inhibits the Ca^2+^ influx in the central nervous system. Aconitine is an important active ingredient in aconitum that acts directly on the sodium ion channel, which not only changes the voltage sensitivity and ion selectivity of a sodium ion channel but also reduces the hyperpolarization potential caused by Na^+^ current activation as well as reduces the maximum inward current (Zhao et al., 2020). In addition, 3-acetylaconitine has been reported to inhibit the excitation through mediation of sodium channels. Tetramethylpyrazine has also been reported to inhibit the calcium channels (Jin et al., 2019).

β-Asarone, which is an effective component of acorus tatarinowii, has been demonstrated to plays neuroprotective roles *in vitro* studies through the inhibition of the Ca^2+^ influx. In addition, α-asarum has been reported to inhibit voltage-gated sodium channels (NAV1.2 channels) (Yuan and Liu., 2020). In summary, most of the natural compounds can be applied to the treatment of epilepsy mainly because they can affect the Ca^2+^ and Na^+^ activities. More natural compounds that regulate ion concentration and ion channels are given in Table 3.

**TABLE 3:** Natural drugs used to treat epilepsy by regulating the concentration of ions.

Natural drugs	Compounds	Chemical structure	Animal models	Mechanisms	References
*Salvia miltiorrhiza Bunge (Lamiaceae)*	Tanshinone IIA	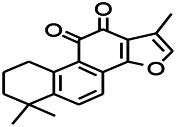	PTZS in rats; 4-APS in mice	Increasing presynaptic Ca^2+^ inflow	Tan et al. (2014); Lin et al. (2013)
*Crataegus pinnatifida Bunge. Var. major (Crataegus L.)*	Hesperidin	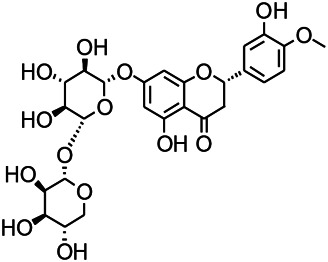	PTZS in mice	Blocking the effects of enhanced Ca^2+^	Xiang et al. (2014)
*Abelmoschus manihot (Linn.) (Malvaceae)*	Isoquercitrin	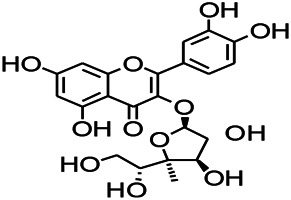	PTZS, MES in mice	Modulating the GABAA-Cl^−^ channel	Xiang et al. (2014)
*Panax ginseng C. A. Meyer (Panax L.)*	Ginsenoside Rb3	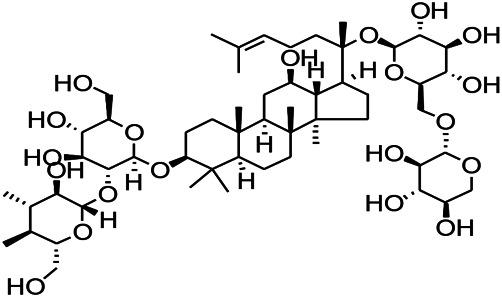	L&PS in rats	Decreasing influx of Ca^2+^	Kim and Rhim (2004)
*Syzygium aromaticum, L. (Syringa oblata Lindl.)*	Eugenol	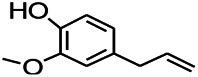	Granule cell	Depressing the transient and late components of Na^+^ in the neurons	Kim (2016)
*Green tea (Camellia sinensis (L.) Kuntze)*	Linalool	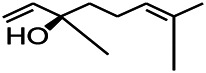	Scn1lab−/− in zebrafish	Improving Na^+^ channels	Kim (2016)
*Portulaca oleracea L. (purslane)*	Baccoside A	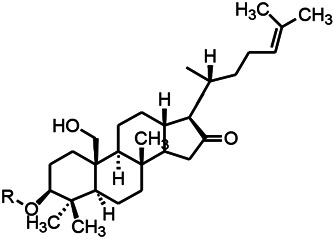	*C. Elegans* at higher temperatures	Regulating T-type calcium channel (CCA-1) protein	Xiang et al. (2014)
*Curculigo orchioides Gaertn. (Hypoxidaceae)*	Citronellol	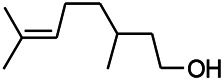	PTXS in mice	Inhibiting neuronal excitability by regulating voltage dependent Na^+^ channels	Xiang et al. (2014)
*Tripterygium wilfordii Hook F.( Celastraceae)*	Triptolide	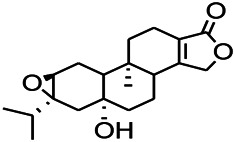	KAS in microglia	Increasing Kv1.1 expression of neurons in hippocampus CA3	Sun et al. (2018); Pan et al. (2012)
*Uncaria rhynchophylla (Miq.) Miq.ex Havil (Rubinaceae)*	Rhynchophylline isorhynchophylline	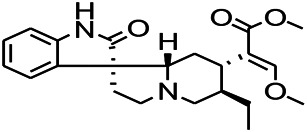 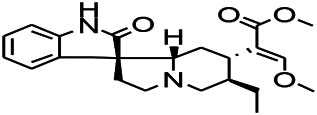	KAS in mice	Decreasing Ca^2+^ internal flow	Ho et al. (2014); Wang and Cai (2018)
*Ziziphus jujuba Mill. var. spinosa (Bunge) Hu ex H. F. Chou (Rhamnaceae)*	Sanjoinine	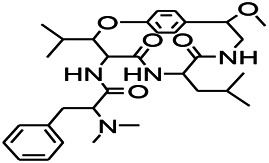	NMDAS in rats	Blocking of intracellular Ca^2+^ influx	Ma et al. (2008)
*Aconitum carmichaeli Debx. (Ranunculaceae)*	Aconitine	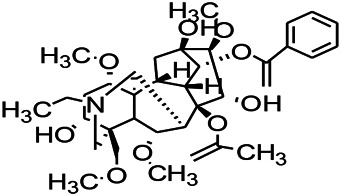	Male Wistar rats	Blocking sodium channels, low Mg^2+^	Zhao et al. (2020)
*Platycodon grandiflorus (Platycodon)*	Nantenine	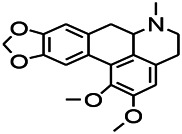	PTZS, MES in mice	Decreasing Ca^2+^ influx into the cell	Xiang et al. (2014)
*Cnidium monnier (L.) Cuss (umbelifera)*	Osthole	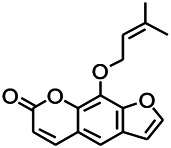	MES in mice	Increasing Kv1.2 expression of neurons in hippocampus CA3	Xiang et al. (2014)
*Acorus tatarinowii Schott (Araceae)*	α-asarone	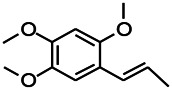	PTZS in mice	Regulating voltage gated sodium ion channel (NAV1.2 channel)	Yuan et al. (2019)
*Acorus tatarinowii Schott (Araceae)*	β-asarone	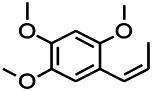	PTZS in mice	Inhibiting of Ca^2+^ influx	Yuan et al. (2019)

4-APS, 4-AP induced seizures; KAS, Kainic acid (KA)-induced seizures; L&PS, Lithium & pilocarpine induced seizure; MES, Maximal electroshock-induced seizures; NMDAS, NMDA induced seizures; PTZS, PTZ-induced seizures; PTXS, Picrotoxin induced seizure.

## Natural Medicines Improves Epilepsy Through the Immune System

Flavonoids have also been often reported to exert immunomodulatory effects. For example, carbenoxolone (CBX) exhibits anti-inflammatory effects through the stimulation of the adrenal glands or by enhancing the effects of endogenous corticosteroids. In a study, the degree of seizure in an epileptic rat was reduced and the incubation period was prolonged after CBX treatment. This effect may be related to the decreased expression of glial fibrillary acidic protein and ligand 43 in cortical rats with epilepsy (Chen et al., 2013). Morin can be extracted from several herbs and fruits. Past studies have demonstrated that morin can reduce the susceptibility to seizures, the expression levels of apoptotic molecules, and the activities of inflammatory cytokines and mammalian target of rapamycin complex 1 (mTORC1) in a seizure model (Lee et al., 2018).

Saponins and glycosides can inhibit inflammation and play an indirect therapeutic role via regulating the content of inflammatory factors. For example, saikosaponin A is an effective monomer extracted from *Radix Bupleuri.* Xie noted that saikosaponin A can dose-dependently decrease the expression of multi-drug resistant protein P-glycoprotein (P-GP) in the temporal cortex and hippocampus, thereby reducing the level of refractory epilepsy caused by pirocarbine (Yu et al., 2012; Xie et al., 2013). Ginsenoside can induce a decrease in the level of IL-1β. Pueraria flavone can regulate the NF-ΚB mRNA and IL-10 mRNA expressions in the hippocampus of epileptic rats (He et al., 2019). Gastrodin can decrease the levels of IL-1β and TNF-α (Yuan et al., 2019). Moreover, ginsenoside possesses the ability of inhibiting microglial cell activation and polarization (Kim and Rhim, 2004).

Some alkaloids have also been reported to possess anti-inflammatory effects. For example, RIN can regulate immune response and neurotrophic factor signaling pathways, such as brain-derived neurotrophin associated with neuronal survival and inflammatory factor IL-1β (Shao et al., 2016). TMP can simultaneously reduce the production of IL-2, IL-6, and TNF-α to counter pentaerythritol-induced epileptic seizures in experimental rats (Liu et al., 2010).

A few coumarins have also been studied *in vivo* in epilepsy patients. For instance, pretreatment of imperata (IMP) has been shown to not only improve the L&PS-induced behavior and memory disorders but also significantly reduce the associated oxidative stress and pain levels. Moreover, it can also reduce the TNF-α and IL-6 levels, leading to significant upregulation of the BDNF levels (Chowdhury et al., 2018).

**FIGURE 2:**
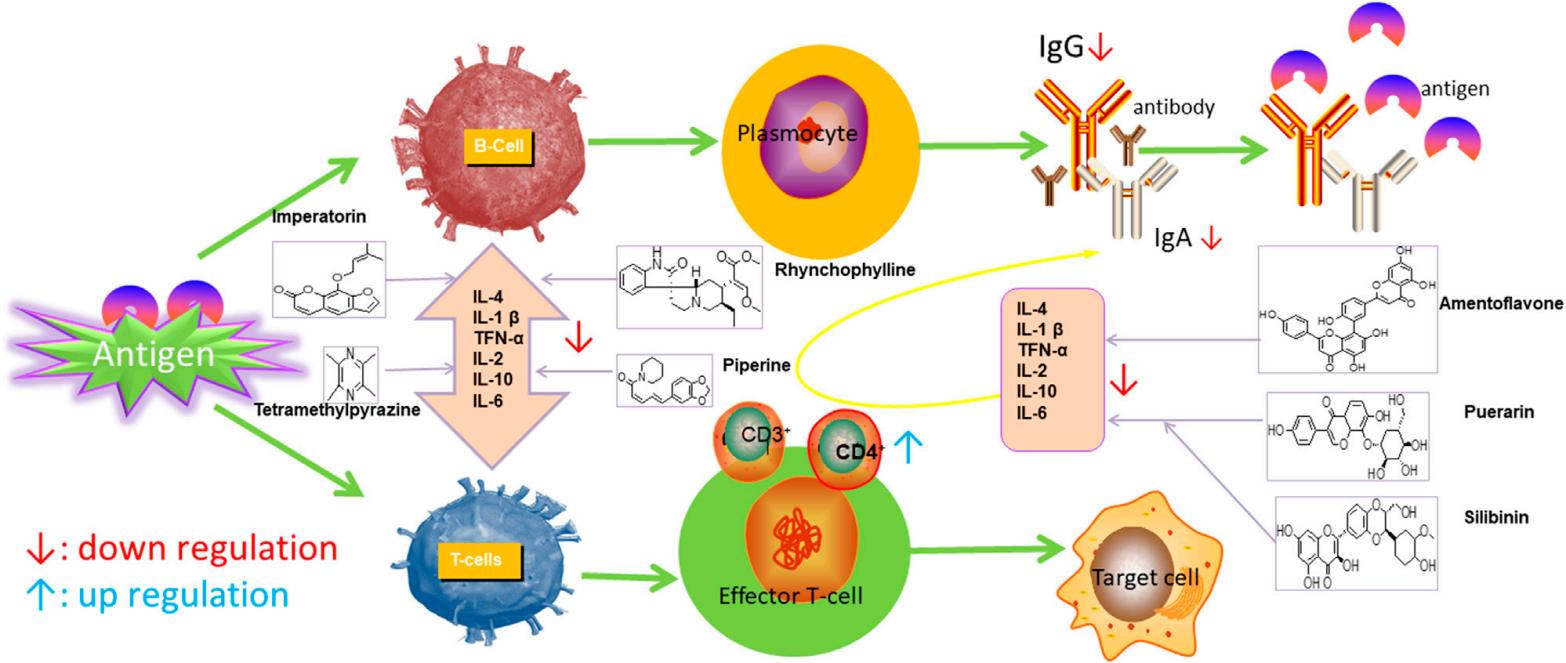
Effects of natural drugs on immune system. Interleukin 1β (IL-1β), interleukin 6 (IL-6), tumor necrosis factor-α (TNF-α).

Ursolic acid (UA), which is found in many human diets and cosmetics, can be used as an antioxidant, anti-inflammatory drugs (Nieoczym et al., 2018). Trans-caryophyllene (TC) applied in epilepsy may be attributed to its ability to reduce the expression of pro-inflammatory cytokines, such as TNF-α and IL-1β (Liu et al., 2015). Several compounds can play a therapeutic role by affecting the immune system, of which flavonoids and saponins are the main components. More natural compounds are displayed in Table 4, and the effects of natural drugs on the immune system is shown in Figure 2.

**TABLE 4:** Natural drugs that treats epilepsy by regulating the immune system and inflammatory factors.

Natural drugs	Compounds	Chemical structure	Animal models	Mechanisms	References
*Radix Puerariae lobatae (Pueraria DC.)*	Puerarin	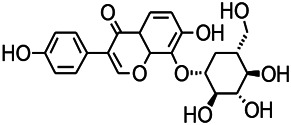	PTZ & PS in rats	Decreasing IL-10	He et al. (2019)
*Nandina domestica Thunb (Berberidaceae)*	Amentoflavone	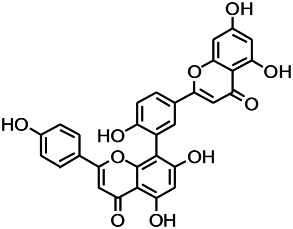	L&PS in rats	Decreasing IL-1β、TNF-α	Zhang et al. (2015)
*Soybean* (*Glycine max (L.)*)	Genistein	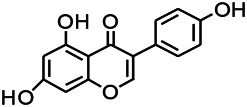	DHPGS in rats	Effecting both cell-mediated and humoral components of the adaptive immune system	Diniz et al. (2015)
*Milk thistle (Silybum marianum)*	Silibinin	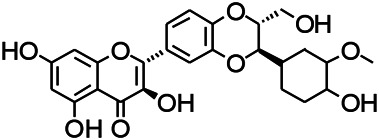	L&PS in mouse	Decreasing TNF-α, IL-1β, and IL-6	Kim et al. (2017)
*Gastrodia elata Blume (*Orchidaceae*)*	Gastrodin	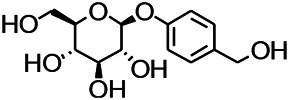	NMDAS in rat	Decreasing, IL-1β and TNF-α	Liu et al. (2018)
*Gardenia jasminoides J. Ellis (Fructus Gardenia)*	Geniposide	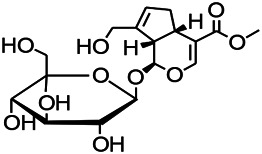	EMS in mouse	Decreasing TNF-a, IL-1β levels and plasma expression of vascular pseudohemophilia factor	Wei et al. (2018)
*Gardenia jasminoides J. Ellis (Fructus Gardenia)*	Ginsenoside Rb3	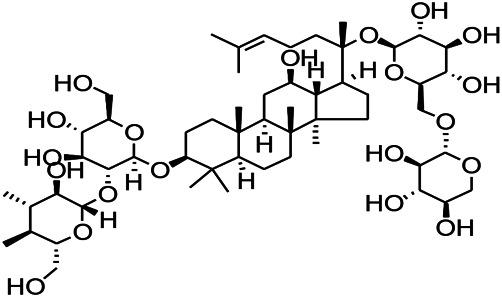	L&PS in rats	Decreasing IL-1β	Kim and Rhim (2004)
*Herbaceous peony (Paeonia lactiflora Pall.)*	Paeoniflorin	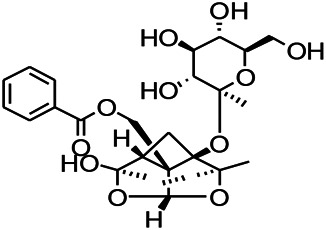	MCC in rats	Inhibiting the inflammatory response; protecting neuronal activity	Hino et al. (2012)
*Crocus sativus L. (saffron)*	Crocin	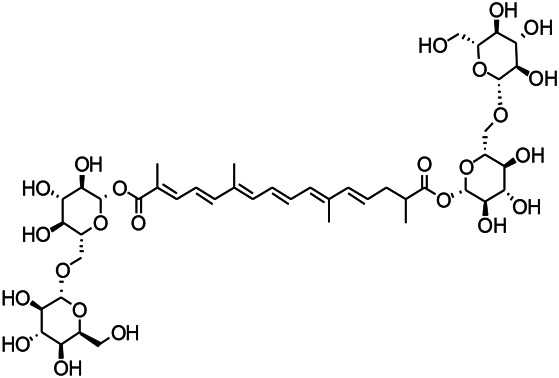	HRKS in mice	Suppressing formation of advanced glycation products and brain inflammatory mediators IL-1β, TNF-α	Mazumder et al. (2017)
*The fragrant camphor tree (Cinnamomum camphora)*	Borneol	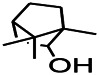	PTZS in mice	Anti-inflammatory; anti-bacterial; protecting central nervous	Tambe et al. (2016)
*Uncaria rhynchophylla(Miq.)Miq.ex Havil (Rubiaceae)*	Rhynchophylline isorhynchophylline	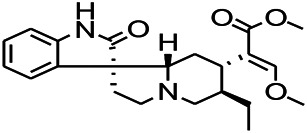 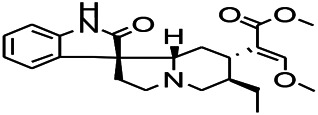	KAS in mice	Decreasing IL-1β	Shao et al. (2016); Wang and Cai (2018)
*Piper nigrum L.(pepper berries)*	Piperine	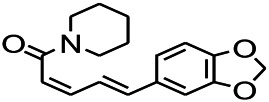	PTZS in both zebrafish and mice	Decreasing TNF-α	Diniz et al. (2015)
*Ligusticum chuanxiong hort (Umbelliferae)*	Tetramethylpyrazine	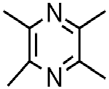	Epileptic Sprague Dawley rats	Decreasing IL-2, IL-6, TNF-α, Bim	Jin et al. (2019)
*Peucedanum praeruptorum Dunn (Radix Peucedani)*	Imperatorin	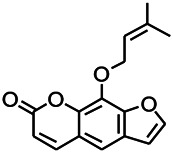	MES in mice	Decreasing TNF-α and IL-6 levels	Chowdhury et al. (2018)

Asp, aspartic acid; 4-APS, 4-AP induced seizures; AS, Audiogenic induced seizures; DHPGS, (S)-3,5-dihydroxyphenylglycine induced models; HRKS, Hippocampus rapid kindling model was established in C57BL/6J; KAS, Kainic acid (KA)-induced seizures; L&PS, Lithium & pilocarpine induced seizure; MES, Maximal electroshock-induced seizures; MCC, Metallic cobalt to the cerebral cortex; NMDAS, NMDA induced seizures; PTZS, PTZ-induced seizures.

## Natural Medicines Improves Epilepsy by Correcting the Glial Cells

Amentoflavone can significantly inhibit the expression of COX2 and iNOS, as well as inhibit the activation of BV-2 microglia (Reng et al., 2020). Carbenoxolone pretreatment or treatment could significantly reduce the connexin expression in the cortex, inhibit glial fibrillary acidic protein expression, and ameliorate the extent of seizure in the experimental rats (Chen et al., 2013). In the persistent epileptic mode induced by pilocarpine, α-asarone inhibited the activation of microglia cells in rat brain tissues (Yuan and Liu, 2020). TL may exert suppressive effects on the expression of major histocompatibility complex class (MHC II) in KA-activated microglia; this mechanism may involve the regulation of the AP-1 activity (Sun et al., 2018). More natural compounds also play a therapeutic role in epilepsy through the regulation of glial cells (Table 5).

**TABLE 5:** Natural drugs used to treat epilepsy by regulating glial cells.

Natural drugs	Compounds	Chemical structure	Animal models	Mechanisms	References
*Nandina domestica Thunb (Berberidaceae)*	Amentoflavone	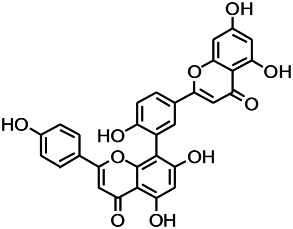	L&PS in rats	Inhibition of microglial activation and reactive proliferation of astrocytes	Reng et al. (2020)
*Glycyrrhiza glabra L. (Fabaceae)*	Carbenoxolone	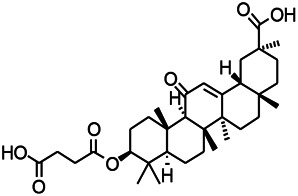	Rat model of ferric ion-induced posttraumatic epilepsy	Reduced cortical glial fibrillary acidic protein and connexin 43 expression	Chen et al. (2013)
*Panax quinquefolius L. (Araliaceae)*	Ginsenoside Rb3	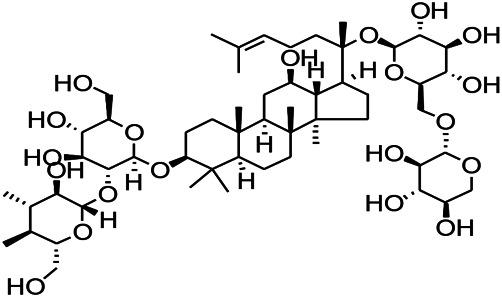	L&PS in rats	Inhibiting microglial cell activation and polarization	Kim and Rhim (2004)
*Tripterygium wilfordii Hook F.(Celastraceae)*	Triptolide	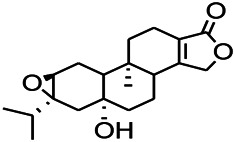	KAS in microglia	Inhabiting expression of IIMHC II	Sun et al. (2018)
*Acorus tatarinowii Schott (Araceae)*	α-asarone	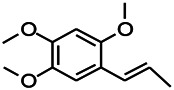	PTZS in rats	Inhibiting microglia cell activation in rat brain tissue	Yuan and Liu (2020)

IIMHC, major histocompatibility complex class; L&PS, Lithium & pilocarpine induced seizure.

## Natural Medicines Improves Epilepsy by Correcting Mitochondrial Dysfunction and Oxidative Stress

The role of flavonoids in epilepsy treatment involves regulating oxidative stress (Diniz et al., 2015), such as glutathione and superoxide dismutase (SOD) (Oliveira et al., 2018). Curcumin, extracted from *Radix curcumae,* reduces the expression of COX-2, 5-lipoxygenase mRNA and protein in the hippocampal neurons of epileptic rats through the regulation of free radicals and carbon monoxide synthase and improves the anti-oxidative stress effect, hippocampal neuron damage, and cognitive function of pilocarp-induced epileptic in experimental rats (Kiasalari et al., 2013). Another study reported that nobiletin can markedly decrease the expression of caspase-3, Bad, Bax, Glu, and PTEN and activate the PI3K/Akt pathway while upregulating the expression of phosphorylated Akt, GSK-3, mTORc-1, and mTORc-2 (Yang et al., 2018).

Glycosides have been reported to exert antioxidant activity. Otophylloside N (OtoN) is one of the ginseng saponins extracted from *Cynanchum otophyllum Schneid*. OtoN can downregulate the Bax/Bcl-2 ratio and increase the level of c-Fos and play an anti-epileptic role (Sheng et al., 2016). Moreover, otophylloside A, B and two c-21 steroidal saponins have also been reported as the main active ingredients for the treatment of epilepsy (Zhao et al., 2013). Ginsenoside, a partially purified extract from *American ginseng*, has been shown to exert anticonvulsant activity. Rb1 can ameliorate cognitive deficits induced by PTZ as well as dose-dependently increase the GSH levels, decrease the MDA levels, and alleviated neuronal injury. In addition, under Mg^2+^-free condition, Rb1 can increase the cell activity and reduce neuronal apoptosis as well as demonstrate a certain dose-dependence mechanism. An *in vitro* and *in vivo* study revealed that Rb1 can also enhance the Nrf2 and HO-1 expressions (Wei et al., 2017). Crocin can significantly increase the activity of SOD, decrease the level of reactive oxygen species (ROS), and reduce the level of nuclear factor-κB (NF-κB) in the hippocampus of PTZ-induced animal models (Mazumder et al., 2017). Gastrodin can increase the expression of CAT, GSH, and SOD (Liu et al., 2018).

In the study on epilepsy, some alkaloids were noted to exert antioxidant activities. For example, RIN was reported to increase the activity of serum SOD. In addition, tricholonin has been reported to reduce the levels of hippocampal mitochondrial MDA and scavenge-free radicals, thereby reducing oxidative stress induced by PTZ kindling.

Terpenoids are mostly detected in volatile oils, which has been reported to demonstrate some antioxidant activities. UA, which is detected in several human diets and cosmetics, can be used as an antioxidant. TC is a component that can be isolated from volatile oils of several flowering plants. Pretreatment of TC can preserve the activity of SOD, GPx, and CAT in the mitochondria as well as reduce the resultant oxidative damage (Liu et al., 2014). After borneol treatment, the levels of SOD, GSH, and CAT in a PTZ-kindling model increases, demonstrating certain antioxidant effects.

**FIGURE 3:**
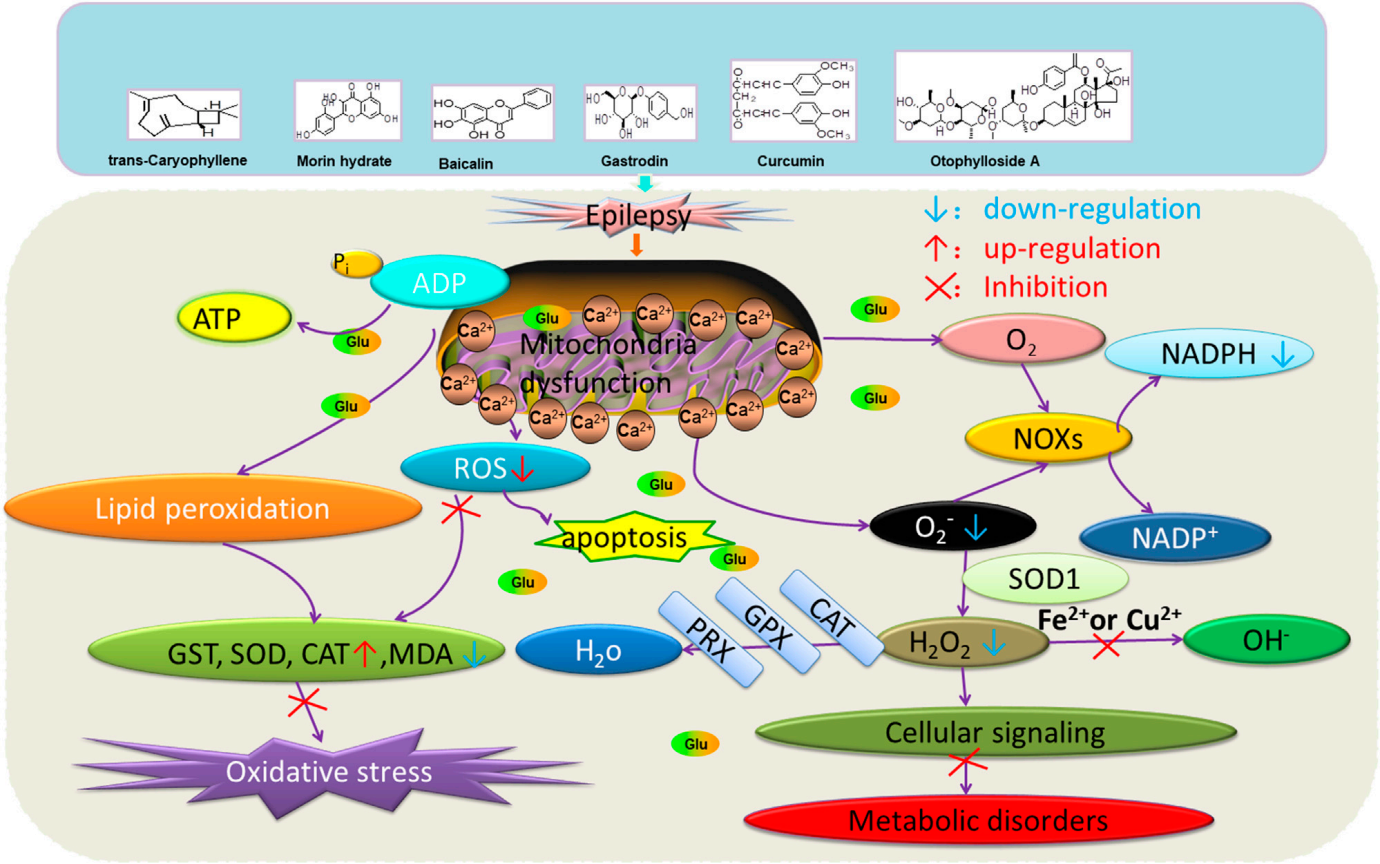
Effects of natural drugs on mitochondrial damage and oxidative stress. Adenosine triphosphate (ATP), adenosine diphosphate (ADP), catalase (CAT), glutathione S-transferase (GST), glutathione peroxidase (GPX), peroxiredoxin (PRX), malondialdehyde (MDA), reactive oxygen species (ROS), superoxide dismutase (SOD), nicotinamide adenine dinucleotide phosphate (NADPH) oxidase (NOXs).

Beta-asarone plays a neuroprotective role through the stabilization of the mitochondrial membrane potential to reduce Glu damage to neurons. α-Asarum reduces the neuronal injury of epilepsy by adjusting the neuron apoptosis factor Bax and the abnormal expression of Bcl-2 (Ma et al., 2011; Wang et al., 2014). More natural compounds also play a therapeutic role in epilepsy through the regulation of SOD or oxidation levels (Table 6). The effects of natural drugs on mitochondrial damage and oxidative stress are illustrated in Figure 3.

**TABLE 6:** Natural drugs used to treat epilepsy by regulating mitochondrial damage and oxidative stress.

Natural drugs	Compounds	Chemical structure	Animal models	Mechanisms	References
*Curcuma longa L. (turmeric)*	Curcumin	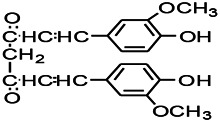	PTZS and KAS in mice	Anti-oxidative stress; Decreasing COX-2,5-LOX, acetylcholinesterase	Kiasalari et al. (2013)
*Radix Puerariae lobatae (Pueraria DC.)*	Puerarin	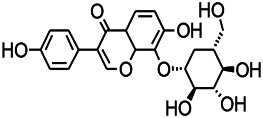	PTZ & PS in rats	Decreasing NF-κB; Antioxidant and anti-apoptotic mechanisms	He et al. (2019)
*Nandina domestica Thunb (Berberidaceae)*	Amentoflavone	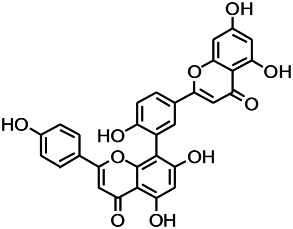	L&PS in rats	Decreasing COX-2, NF-κB p65	Zhang et al. (2015)
Green tea (Camellia sinensis (L.) Kuntze)	Catechin	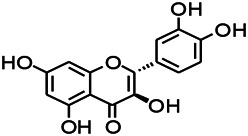	PTZS in rats	Ameliorating cognitive impairment and oxidative stress	Ahmad et al. (2020)
*Eclipta prostrata L. (Asteraceae)*	Luteolin	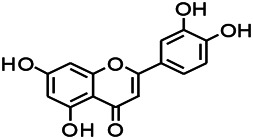	PTZS in mice	Inhibiting oxidative stress	Tambe et al. (2016)
*Green tea* (*Camellia sinensis (L.) Kuntze*)	EGCG	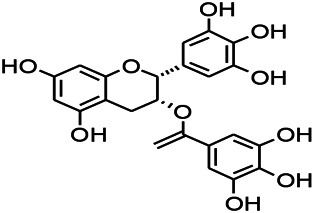	L&PS in rats	Inhibiting TLR4, NF-κB signaling pathway; Antioxidant	Qu et al. (2019)
*Milk thistle (Silybum marianum)*	Silibinin	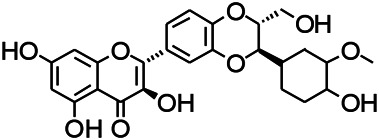	L&PS in mouse	Decreasing Hif-1α	Kim et al. (2019)
*Chlorophora tinctoria (L.)*	Morin hydrate	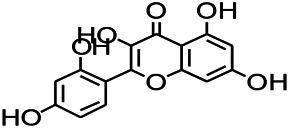	PTZS in mouse	Modulating the concentrations Na^+^/K^+^-ATP; Antioxidant status	Lee et al. (2018)
*Astragalus spp. (Radix Astragali)*	Baicalin	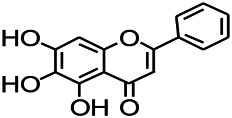	PTZS in rats	Decreasing Bcl-2, GSH, SOD, IL-1β, Bax, caspase-3, TNF-α, Lipid peroxidation, nitrite	Diniz et al. (2015)
*Citrus reticulata Blanco (Rutaceae)*	Nobiletin	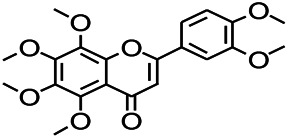	PTZS in mice	Antiapoptotic	Yang et al. (2018)
*Smoke tree (Cotinus coggygria)*	Fisetin	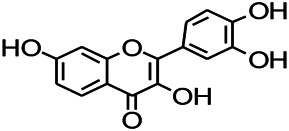	Iron-induced experimental model in rats	Inhibiting oxidative injury	Diniz et al. (2015)
*Gastrodia elata Blume (Orchidaceae)*	Gastrodin	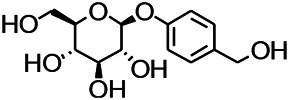	NMDAS in rat	Increasing CAT, GSH, SOD	Liu et al. (2018)
*Cynanchum otophyllum Schneid (Asclepiadaceae)*	Otophylloside	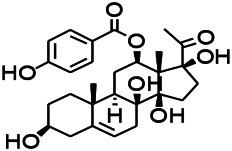	PTZS in mice	Downregulating Bax/Bcl-2 ratio; Increasing the expression level of c-fos	Sheng et al. (2016)
*Panax quinquefolius L. (Araliaceae)*	Rb1 ginsenosides	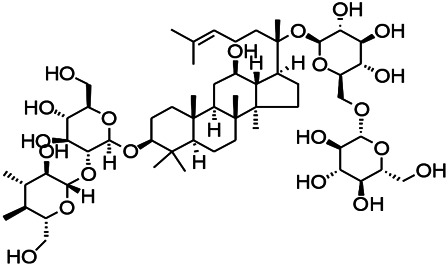	PTZS in rat	Increased GSH levels, decreased MDA levels, enhanced both the Nrf2 and HO-1 expressions	Shi et al. (2018)
*Syringa oblata Lindl.(Oleaceae)*	*Trans*-Caryophyllene	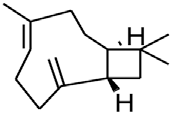	KAS in mice	Preserving the activity of gpx, SOD, and CAT	Liu et al. (2015)
*Strawberries (Fragaria X ananassa, Duch.)*	Γ-Decanolactone	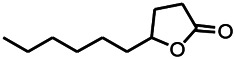	PTZS in mice	Protecting oxidative stress and DNA damage in mice	Xiang et al. (2014)
*Eucalyptus citriodora Hook. (Myrtaceae)*	Isopulegol	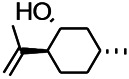	PTZS in mice	Decreasing in lipid peroxidation; preserving catalase activity in normal levels; preventing loss of GSH	Xiang et al. (2014)
*Acorus tatarinowii Schott (Araceae)*	α-asarone	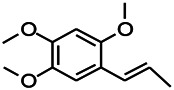	PTZS in mice	Regulating the abnormal expression of neuronal apoptotic factors Bax and Bcl-2	Liu et al. (2013)
*Acorus tatarinowii Schott (Araceae)*	β-asarone	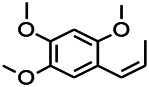	PTZS in mice	Stabilizating mitochondrial membrane potential	Yuan et al. (2019)

KAS, Kainic acid (KA)-induced seizures; L&PS, Lithium & pilocarpine induced seizure; NMDAS, NMDA induced seizures; PTZS, PTZ-induced seizures.

## Natural Medicines for Epilepsy Treatment Through Other Mechanisms

Several natural compounds have been reported with antiepileptic effects, and their mechanisms are complex. Silibinin, the main active ingredient isolated from *Silybum marianum (L.)*, has been reported to administrate dramatically inhibited KA-induced GCD and mTORC1 activation, while the phosphorylation levels of the mTORC1 substrate 4E-BP1 and p70S6K were also altered (Kim et al., 2017).

Terpenoids can also play a therapeutic role through other mechanisms. UA, which is detected in several human dietic components and cosmetics, can be used as an antioxidant, anti-inflammatory, antibacterial, and anti-tumor agent. In addition, its anticonvulsant effects were demonstrated in the 6-Hz-induced psychomotor seizure threshold test. This effect was further validated in the maximum electroconvulsive threshold test and time-sharing intravenous injection of pentaerythrazol (Nieoczym et al., 2018). Pathological studies indicated that borneol had a neuroprotective effect at an appropriate dose, which was manifested as decreased GFAP level on immunostaining (Tambe et al., 2016).

Alkaloids are also widely applied in the treatment of epilepsy. RIN downregulate the expression of TLR4 in hippocampal tissues and exert a protective effect on the brain injury of rats caused by the persistent state of convulsion (Wang and Cai, 2018). Mesaconitine (MA) can also reduce the excitability by inhibiting the norepinephrine uptake [3H] within a certain concentration range. TMP can enhance the expression of adhesion molecule-140 in hippocampal neurons and reduce the expression of apoptotic factor Bim, thereby playing a protective role on the neurons (Yu et al., 2010; Fang and Zhang, 2013).

The synergistic effect of xanthotoxin combined with oxacipine and topiramate in the maximum electroshock-induced epilepsy test suggests that xanthocyanin may play a role similar to that of an antiepileptic drug at certain level. However, there are only limited data available on this aspect, thus requiring further verification of this hypothesis (Zagaja et al., 2016).

Gardenoside is one of the main active components of *G. jasminoides Ellis*, and past studies have demonstrated its applicability in treating epilepsy. The main mechanism of treatment may be to reduce the expression of COX-2 and AP-1 and regulate the apoptosis of nerve cells by the PI3K/Akt/GSK-3 signaling pathway (Wei et al., 2017).

Subtilisli A has been reported to exert an antiepileptic effect through the mediation of rho-activated coiled-coil kinase. In addition, it can regulate neurotransmitters and apoptotic factors. Moreover, α-asarum plays a protective role in cases of treatment-resistant epilepsy that induced neuronal cell membrane damage through the inhibition of the laminin-1 expression (Huang et al., 2013). Other compounds have been found to exert antiepileptic effects in pharmacological studies, such as umbelliferone (UMB; Zagaja et al., 2015), erysothrine (Rosa et al., 2012), resveratrol, and catechin (Ahmad et al., 2020). In addition, the anticonvulsant and epileptic effects of some protein components have also been studied. Some other natural compounds are shown in Table 7.

**TABLE 7:** he other.

Natural drugs	Compounds	Chemical structure	Animal models	Mechanisms	References
*Curcuma longa L. (turmeric)*	Curcumin	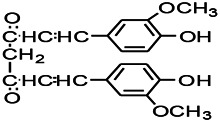	PTZS and KAS in mice	Inhibit MTORc1 activation; Reduce the damage of hippocampal neurons and cognitive dysfunction	Kiasalari et al. (2013)
*Citrus aurantium L. (Rutaceae)*	Naringin	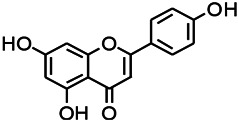	KAS in mice	Reducing GCD and mtorc1 activation	Diniz et al. (2015)
*Sophora japonica L. (Fabaceae)*	Rutin	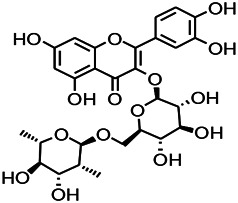	PTZS in zebrafish; KAS in mice	Improving epileptoid action	Diniz et al. (2015)
*Folium Sennae (Senna)*	Vitexin	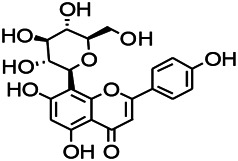	PTZ-CS	Neuroprotective effects	Diniz et al. (2015)
*Achillea millefolium L. (Yarrow)*	Kaempferol	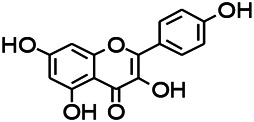	Epileptic *drosophila*	Inhibition of DNA topoisomerase I enzyme	Diniz et al. (2015)
*Smoke tree (Cotinus coggygria)*	Fisetin	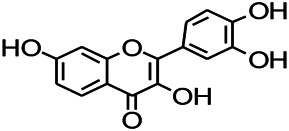	Iron-induced experimental model in rats	Protecting endogenous enzyme level	Diniz et al. (2015)
*Dendranthema morifolium (Ramat.) Tzvelev*	Linarin	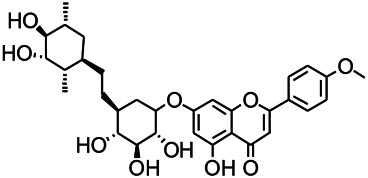	PTZS in mice	Preventing CNS excitation or stress	Diniz et al. (2015)
*Cannabis sativa L. (Moraceae, hemp)*	Cannabidiol	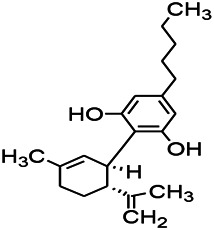	Patients	Inhabiting neuronal excitability	Xiang et al. (2014)
*Herba Menthae Haplocalycis (Mentha haplocalyx Briq.)*	Carvone	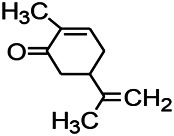	PTZS in mice	Inhibiting central nervous system	Xiang et al. (2014)
*Origanum vulgare L. (Lamiaceae Martinov)*	Γ-terpinene	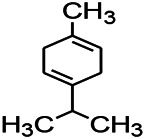	PTZS, MES in mice	Raising the threshold of convulsion	Xiang et al. (2014)
*Norway spruce* (*Picea abies (L.) Karst.*) *trees*	Verbenone	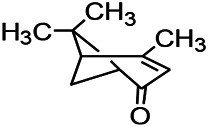	PTZS in mice	Related to RNA expression of COX-2, BDNF, and C-fos	Bahr et al. (2019)
*Cannabis sativa (marihuana)*	Delta9-tetrahydrocannabinol	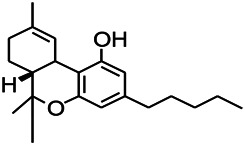	PTZS in mice	Improving seizures in children	Bahr et al. (2019)
*Nepeta cataria L. var. citriodora (Lamiaceae)*	Ursolic acid	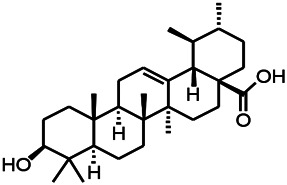	PTZS in mice	Unknown	Nieoczym et al. (2018)
*Moschus moschiferus L*	Muscone	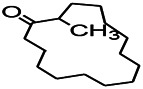	Rat	Inhibiting the central nervous system excitability	Bahr et al. (2019)
*The fragrant camphor tree (Cinnamomum camphora)*	Borneol	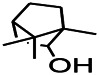	PTZS in mice	Anti-bacterial; protecting central nervous	Tambe et al. (2016)
*Aconitum carmichaeli Debx. (Ranunculaceae)*	Mesaconitine	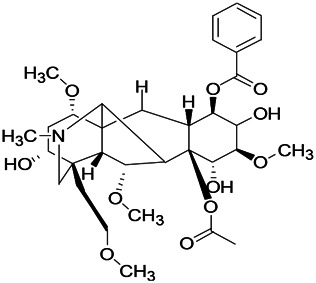	Wistar rats	Regulating the noradrenergic system	Zhao et al. (2020)
*Rhizoma Corydalis (Papaveraceae)*	Dl-Tetrahydropalmatine	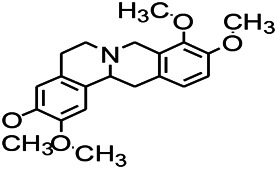	Electrical kindling in rats	Reducing dopamine output	Lin et al. (2002)
*Rauwolfia serpentine (Sarpagandha)*	Raubasine	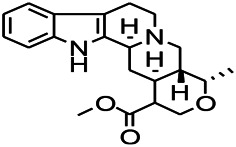	PTZS in mice	Interacting at benzodiazepine sites with a benzodiazepine agonist-type activity	Xiang et al. (2014)
*Piper nigrum L. (Pepper berries)*	Piperlongumine	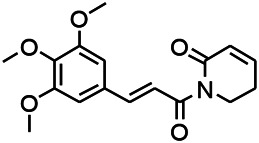	PTZS in mice	Decreasing the latency to death in mice	Xiang et al. (2014)
*Erythrina mulungu Mart ex Benth (Papilionaceae)*	Erythravine	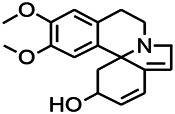	BCLS in Wistar rats	Unclear	Xiang et al. (2014)
*Ligusticum chuanxiong hort (Umbelliferae)*	Tetramethylpyrazine	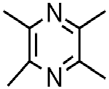	Epileptic Sprague Dawley rats	Increasing neuron cell adhesion molecule -140	Jin et al. (2019)
*Peucedanum praeruptorum dunn (Umbelliferae)*	Imperatorin	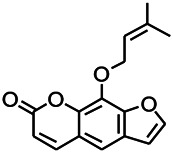	MES in mice	Upregulating of BDNF levels	Chowdhury et al. (2018)
*Heracleum mantegazzianum s.l. (Giant hogweed)*	Umbelliferone	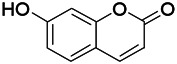	MES in mice	Unclear	Zagaja et al. (2015)
*Zanthoxylum schinifolium Sieb. et Zucc. (Rutaceae)*	Xanthotoxin	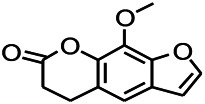	MES in mice	Unclear	Zagaja et al. (2016)
*Acorus tatarinowii Schott (Araceae)*	α-asarum	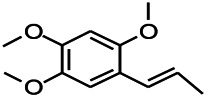	Caco -2 cells	Decreased expression of P glycoprotein and multidrug resistance gene	Yuan and Liu. (2020)

BCLS, bicuculline induced seizure; KAS, Kainic acid (KA)-induced seizures; MES, Maximal electroshock-induced seizures; PTZS, PTZ-induced seizures; PTZ-CS, PTZ induced chronic seizures.

## Combined Use of Natural Medicines and Anti-Epileptic Conventional Drugs

Drug therapy remains the dominant mode of epilepsy control. The efficacy of Western medicine in epilepsy control is clear, with several known adverse reactions, such as anorexia, damaged liver function, dizziness, headache, decrease of white blood cells, cognitive function decline, and a decrease in life quality (Zhu et al., 2017). Especially for peadetric patients, the physical damage through these Western medicine is even greater (Tang et al., 2016). On the other hand, natural drugs have little toxic and side effects with lesser discomfort to patients than that by Western drugs. In the recent years, past studies have recorded that the combination of conventional Chinese and Western medicine can bring hope to epilepsy patients who cannot be otherwise treated with Western medicine (Qu and Zhang, 2019). Moreover, conventional Chinese medicine and the conventional Chinese medicine prescriptions can effectively improve the efficacy of Western medicine as well as effectively reduce the adverse reactions resultant from the usage of Western medicine (Huang et al., 2013). Chinese medicine in the remission of conditioning tonic enhances patients’ anti-epilepsy and anti-convulsion states, reduces the nerve damage in patients during the course of epilepsy, and make patients more conducive to the recovery from the disease.

### Nobiletin and Clonazepam

Nobiletin and clonazepam significantly reduce seizure severity. The administration of clonazepam and nobiletin can downregulate seizure-induced increases in apoptotic protein expression and apoptotic cell count, restore the Glu/GABA balance, and modulate the expression of GABAA and GAD 65. Moreover, the administration of nobiletin and clonazepam can significantly upregulate the phosphoinositide 3-kinase/protein kinase B (PI3K/Akt) signaling (Yang et al., 2018).

### UMB, Valproate, and Phenobarbital

Systemic intraperitoneal (ip) administration of UMB at a dosage of 150 mg/kg could significantly elevate the threshold for EMS in mice. The selective potentiation of the anticonvulsant potency of phenobarbital and valproate by UMB and the lack of any pharmacokinetic interactions between the drugs make the combinations of UMB with phenobarbital or valproate worthy of consideration for refractory epileptic patients (Zagaja et al., 2015).

### Naringin and Phenytoin

Naringin in combination with phenytoin has demonstrated a protective effect against seizures as well as improved the conditioned avoidance response in PTZ-induced kindling model. This combination can improve the neurochemical balance by elevating the levels of GABA and dopamine, decrease the levels of Glu and MDA, and increase the levels of antioxidants GSH, SOD, CAT, and total thiol. Therefore, the co-administration of naringin with phenytoin offers a potential treatment option for epilepsy (Phani et al., 2018).

### Gastrodin and Carbamazepine

Gastrodin combined with carbazepine can improve the treatment progression of epilepsy patients with a significant clinical efficacy as well as improve the electroencephalogram abnormalities and the overall treatment effects (Guo, 2017), with fewer complications, which is cumulatively conducive to improve the prognosis of patients and their quality of life (Liu et al., 2018).

### Compound Preparation

**FIGURE 4:**
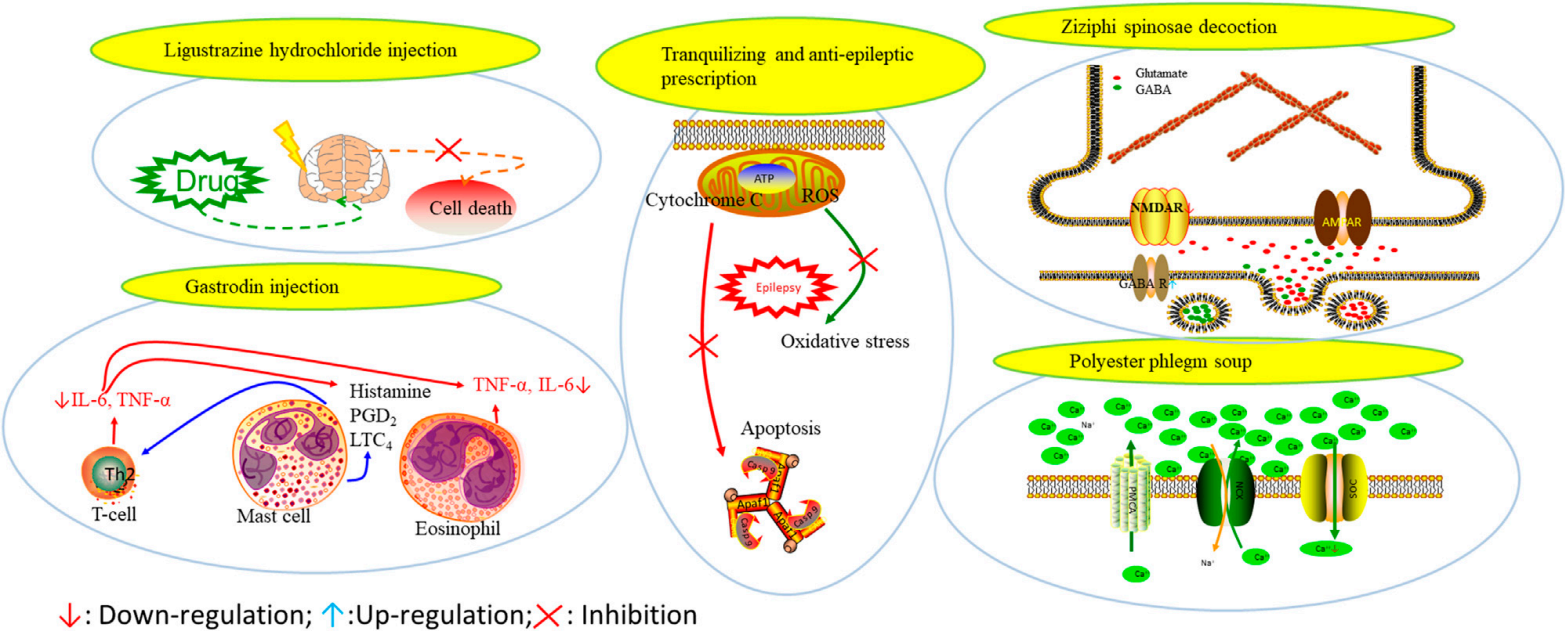
The therapeutic mechanism of integrated Chinese and Western medicine. Interleukin 6 (IL-6), reactive oxygen species (ROS), tumor necrosis factor-α (TNF-α), prostaglandin D2 (PGD2), leukotriene C4 (LTC4), γ-aminobutyric acid (GABA).

Prescriptions are more widely used in the clinical practice, and the resultant therapeutic effect is also recognized by more patients (Tian, 2015; Yang et al., 2015). The right combination of two drugs can not only reduce the toxicity and enhance the efficacy of a single drug use but also provide a more pleasant treatment experience to the patients (Roseti et al., 2015). For example, ziziphi spinosae decoction (*Ligusticum chuanxiong hort*, *Glycyrrhiza glabra L*, *Semen ziziphi spinosae*, *Anemarrhena asphodeloides Bunge*) decreased has been reported to decrease the expression of glu and NMDAR1 (Lu et al., 2020). In addition, polyester phlegm soup (*Curcuma rcenyujin Y*, *H. Chenet C. Ling*, *Ligusticum chuanxiong hort*, *Angelica sinensis (Oliv.) Diels*, *Prunus persica (L.) Batsch*, *Pinellia ternata (Thunb.) Breit*, *Paeonia lactiflora Pall*, *Pericarpium citri reticulatae*, and *Carthamus tinctorius L.*) has been reported to decrease the levels of Na^+^ and Ca^2+^ (Li, 2018). Moreover, Tongqiao Dingxian Soup (*Bombyx Batryticatus, Agkistrodon, Gastrodia elata Bl, Polygala tenuifolia Willd, Acorus tatarinowii Schott, Pheretima, Androctonus crassicauda, Scolopendridae, Albizia julibrissin Durazz*, and *Gardenia jasminoides Ellis*) can reportedly decrease the serum neuropeptide Y, BDNF, and glial fibrino acid protein (Zhou, 2018). Several types of drugs play different roles in the body simultaneously; this special approach of compatibility has achieved the effect of enhancing the curative effect and reducing the toxic and side-effects, for example, with the use of drugs such as ligustrazine hydrochloride injection, tranquilizing and antiepileptic prescription, wild jujube seed decoction, and gastrodin injection. These proprietary drugs have been widely applied, and their therapeutic mechanisms are displayed in Figure 4.

In the past, studies have been performed on the “Dictionary of Traditional Chinese Medicine Prescriptions” for the treatment of epilepsy 532 prescription law analysis (Wu and Zhao, 2017). The drugs with a single drug use frequency of >15% included *Cinnabaris, Glycyrrhiza glabra L, Panax ginseng C. A. Meyer, Calculus bovis, Moschus, Polygala tenuifolia Willd, Rheum officinale Baill, Poria cocos(Schw.) Wolf, Rehmannia glutinosa (Gaertn.) DC, Scutellaria baicalensis Georgi*, and *Aconitum carmichaeli Debx*. The drugs are mainly used to calm the liver and quench the wind, fill the deficiency and calm the mind, clear the heat and dissolve phlegm, and open the body to awaken the mind, which are all suitable for the etiology, pathogenesis, and treatment of epilepsy. Based on the analysis of drugs, the most frequently used anti-spasmotic drugs for calming the liver wind include *Calculus bovis, Gastrodia elata Bl, Bombyx Batryticatus, Androctonus crassicauda*, and *Paeonia lactiflora Pall.* The most common drugs for deficiency include qi tonic, blood tonic, and Yin tonic. The representative drugs include *Glycyrrhiza glabra L, Panax ginseng C. A. Meyer, Angelica sinensis (Oliv.) Diels, Paeonia lactiflora Pall*, and *Ophiopogon japonicus (Linn. f.) Ker-Gawl.* The main tranquilizers are the important and mind-nourishing ones. The representative tranquilizers include *Cinnabaris, Dens draconis*, *Polygala tenuifolia Willd*, and *Semen ziziphi spinosae.* Heat-clearing drugs mainly include the heat-clearing and dampness drugs, the heat-clearing and purging gunpowder, and the heat-clearing and blood-cooling drugs; the representative drugs include *Radix scutellariae, Rhizoma coptidis, Gypsum fibrosum*, and *Radix Rehmanniae.* The frequency of *Moschus* was the highest among the prescription drugs. Phlegm-reducing drugs are mainly to warm cold phlegm drugs; the representative drugs include *Rehmannia glutinosa (Gaertn.) DC and Arisaema heterophyllum Blume*.

## Other Forms of Epilepsy Treatment

Nursing intervention (Dong, 2018) and neural stem cell transplantation has also been gaining increasing attention as a treatment approach (Thodeson et al., 2018). In addition, there is an extremely interesting report that regular consumption of bacopa monniera tea can improve the epilepsy condition as well as alleviate dementia, psychosis, and other neurological disorders, which may be linked to improved learning and reasoning abilities among adults and children (Krishnakumar et al., 2009). In addition, based on the epileptic comorbid embarks, the new guideline on the exploration of epileptic treatment can be regarded as a new way. Among these, the GABA receptor pathway plays an important role in various mental diseases (Johnston, 2015). We have summarized here the drugs utilized that showed GABA excitatory effects (Table 8).

**TABLE 8:** A monomer compound derived from a natural drug that modulated ionotropic GABA receptors.

Compound	Structure	Application	References
Malvidin	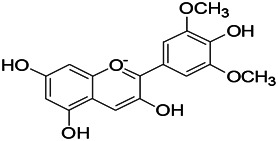	Osteoarthritis, Premature senescence, Myocardial infarction	Johnston (2015)
Taxifolin	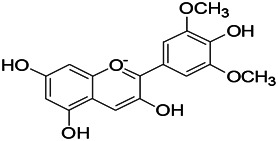	Osteoclastogenesis, Antioxidant, Alzheimer’s disease, hyperuricemic	Johnston (2015)
Agathisflavone	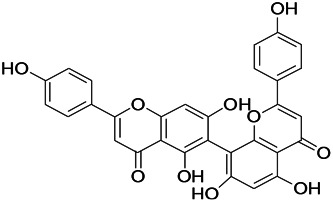	Protecting nerve; anti-oxidative damage and anti-viral	Shrestha et al. (2012)
2-Ethyl-7-hydroxy-3′,4′-methylenedioxy-6-propylisoflavone	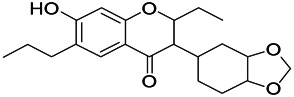	Unclear	Hanrahan et al. (2015)
Glabrol	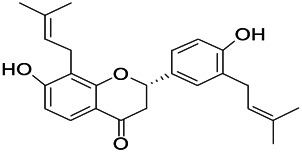	Antibacterial	Hanrahan et al. (2015)
Glabridin	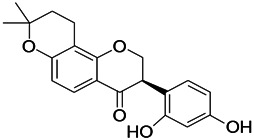	Muscle atrophy, Cardiovascular	Hanrahan et al. (2015)
6-methoxyflavone	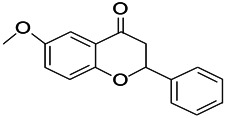	Neuropathic allodynia and hypoalgesia, anti-inflammatory	Hall et al. (2014)
Vicenin	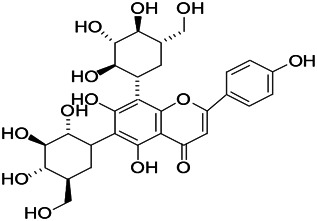	Prostate cancer, vascular inflammation, Radiation protection	Oliveira et al. (2018)
Isovitexin	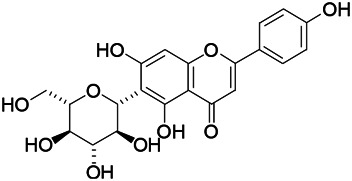	Various activities, such as anti-oxidant [12], anti-inflammatory, anti-AD effects	Oliveira et al. (2018)
Isoliquiritigenin	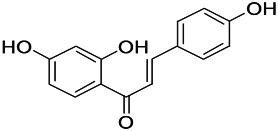	Cervical cancer, breast cancer, hepatoma cancer, colon cancer, prostate cancer, human leukemia, oral carcinoma, Cardioprotective, Hepatoprotective, Antiangiogenic, anti-microbial, anti-anorexia	Wasowski and Marder (2012)
Dihydromyricetin	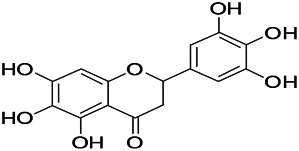	Antioxidant, anti-inflammatory, protecting cells, regulating lipid and glucose metabolism	Wasowski and Marder (2012)
Eriodictyol	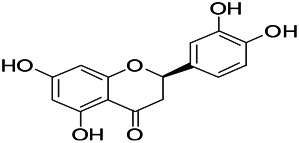	Antioxidant, anti-inflammatory, anti-cancer, neuroprotective, cardioprotective, anti-diabetic, anti-obesity, hepatoprotective, and miscellaneous	Wasowski and Marder (2012)
Hesperetin	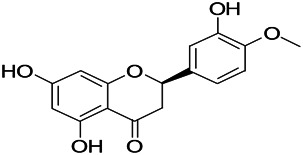	*Cancer*, anti-inflammatory, cataracts, antioxidant and anti-inflammatory	Wasowski and Marder (2012)
6-methylflavanone	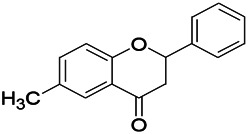	Antioxidant and anticancer	Wasowski and Marder (2012)
3,7-Dihydroxyisoflavan	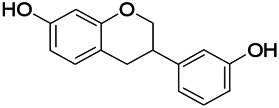	Anti-oxidant, anti-estrogenic, anti-cancerous and anti-inflammatory	Wasowski and Marder (2012)
Oroxylin A	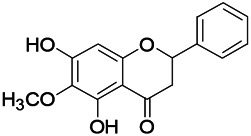	Anti-cancer, antiinflammation, neuroprotective, anti-coagulation	Wasowski and Marder (2012)
2,5,7,-trihydroxy-6,8-dimethoxyflavone	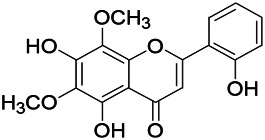	Antioxidant	Wasowski and Marder (2012)
6-methylapigenin	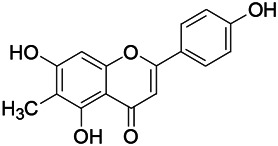	Sedative, sleep-enhancing properties, anxiolytic	Nilsson and Sterner (2011)
Skrofulein	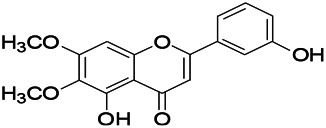	Anti-proliferative, anti-cancer, anti-inflammatory, breast cancer, antioxidative and antiplatelet	Nilsson and Sterner (2011)
Daidzein	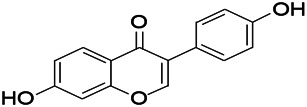	Against various neuropathological conditions mainly by its interaction with the cerebrovascular system, anti-tumor, inhibits choriocarcinoma proliferation	Nilsson and Sterner (2011)
Honokiol	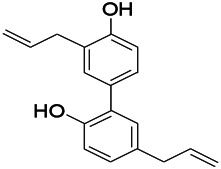	Antioxidant, alzheimer, anticancer, antineoplastic, anti-inflammatory	Nilsson and Sterner (2011)
Magnolol	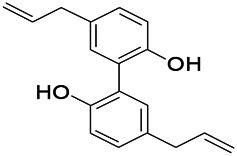	Anti-inflammatory, inflammatory bowel disease, Lung cancer, Hepatocellular carcinoma, Antidepressant	Nilsson and Sterner (2011)
Miltirone	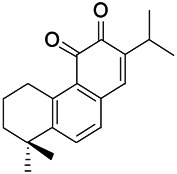	Antileukemic, anti-inflammatory, anti-platelet, resistant lung cancer	Nilsson and Sterner (2011)
Rhusflavone	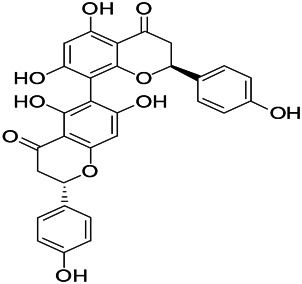	Inducing sleep	Shen et al. (2016)
Galdosol	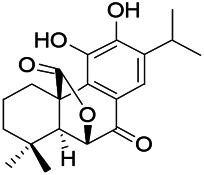	Antioxidant, Cytostatic and antibacterial	Nilsson and Sterner (2011)
Carnosic acid	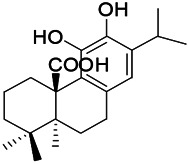	Antioxidant, anti-inflammatory, anticancer activities, antimicrobial, protecting mitochondria	Nilsson and Sterner (2011)
Carnosol	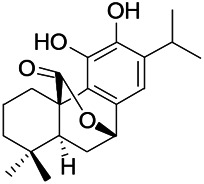	Anti-carcinogenic, atopic dermatitis, gastric tumor, chronic stress, improved lifespan, healthspan	Nilsson and Sterner (2011)
Valerenic acid	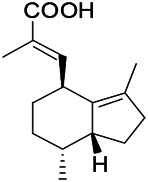	Anxiety, sleep disturbances, postpartum blues, depression, anti-inflammatory	Nilsson and Sterner (2011)
Valeranone	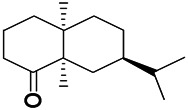	Sedative, tranquilizing antihypertensive properties, hyperkinetic behavior disorders	Nilsson and Sterner (2011)
Valtrate	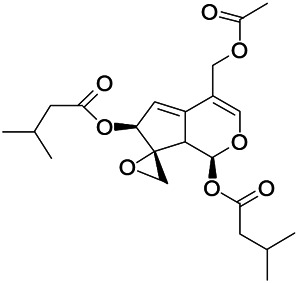	Breast cancer, ovarian cancer, anti-HIV	Nilsson and Sterner (2011)
Baldrinal	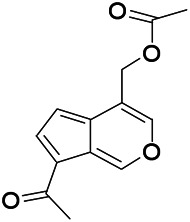	Anticonvulsant effects	Nilsson and Sterner (2011)
(-)-α-thujone	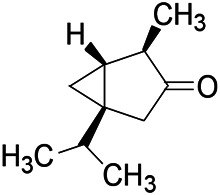	Polycystic ovary syndrome, malignant glioblastoma, Pro-apoptotic and anti-angiogenic	Nilsson and Sterner (2011)
Thymol	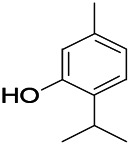	Antibacterial, antifungal, anticancer, Dermanyssus gallinae, anthelmintic, antioxidant, against geotrichum citri-aurantii	Nilsson and Sterner (2011)
Bilobalide	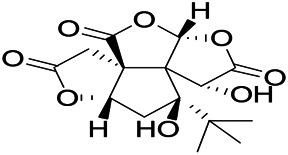	Gastric ulcer, abates inflammation, insulin resistance and secretion of angiogenic factors, alleviates depression	Nilsson and Sterner (2011)
Ginkgolide B	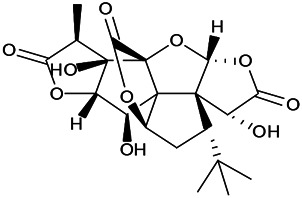	Ischemic stroke, anti-inflammatory and chondroprotective	Nilsson and Sterner (2011)
Isocurcumenol	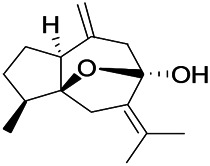	Antitumour, anti-androgenic	Nilsson and Sterner (2011)
6-hydroxyflavone	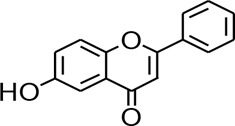	Anxiolytic	Ren et al. (2010)

## Conclusion

Based on our cumulative review, it seems that the occurrence of epilepsy is not only related to the nervous system but also to the body’s immune and metabolic systems, for instance, neurodegenerative protein accumulation, neurotransmitter imbalance, glial cell proliferation, nerve excitability, synaptic changes, neurons voltage, ion channel mutations or variants of ligand, inflammatory reaction, oxidative stress, mitochondria damage, and glycogen metabolism disorders. Natural medicines are precious resources that promises establishment of reliable candidate drugs with low toxicity and presents several effective monomers that possess specific pharmacological activities; these natural medicines include such as artemisinin (Tu, 2016), emodin (Zhang Q et al., 2020), and berberine (Wang et al., 2019). In addition, it has been reported that natural medicines are beneficial in controlling the manifestations of psychiatric disorders (Zhang Y et al., 2020). According to the pathogenesis and symptoms of patients with epilepsy, different ways of treatment have been considered. The outcomes with the use of natural medicine has been superior in this respect. Natural drugs are thus believed to improve the release of neurotransmitters and in relation to the synaptic structure and functions, in the improvement of the imbalance of ion channels, reduction in the release of inflammatory factors, and enhancement in the activity of antioxidant enzymes and in modulating immune responses. Most natural drugs possess therapeutic effects through the regulation of inhibitory neurotransmitters, and anti-inflammatory and oxidative stress pathways, while some drugs have multiple pathways rather than a single target. Moreover, the most surprising aspect is that the combination of natural medicine and western medicine can not only reduce the potential side-effects but also improve the overall therapeutic effect. The combined use of natural medicine and western medicine thus plays a synergistic role with better outcomes (Guo et al., 2015; Kang et al., 2015; Yang et al., 2015). For several patients, a comprehensive treatment approach is thus a reasonable and effective treatment option.

We noted some limitation in the study. For instance, epilepsy pathogenesis research is mostly limited to some animal models. In addition, whether the data from the epilepsy model are directly applicable to humans is also questionable. The pathogenesis of epilepsy is quite complex, and the model of epilepsy induced by a single drug cannot completely simulate the pathogenesis of epilepsy, which brings its own set of difficulties to the treatment approach of epilepsy. Moreover, which of the conclusions drawn from the epileptic model can form the basis of the occurrence of epilepsy requires further comprehension. Therefore, on one hand, it has been suggested that scholars develop new and more vivid comprehensive epilepsy model so as to achieve the same effect of drug application in an epilepsy model and clinical patients. Future studies thus suggest that a combination of clinical practice and theory should be considered for such cases. On the other hand, gene therapy as a new treatment should attract academic attention. The theory that changes in a single gene can induce epilepsy needs, requiring further verification to enable future research to focus on the whole genome. In addition, neural stem cell transplantation, as a new technique, has achieved good outcomes in the field of epilepsy.
