# Supplementary material for: Natural Medicines for the Treatment of Epilepsy: Bioactive Components, Pharmacology and Mechanism
Source: Front Pharmacol. 2021 Mar 4;12:604040. doi: 10.3389/fphar.2021.604040 (PMC7969896; doi:10.3389/fphar.2021.604040)
Supplement: Supplementary file 1 [file datasheet1.pdf]

# *Supplementary Material*

## 1.1 Supplementary Figures

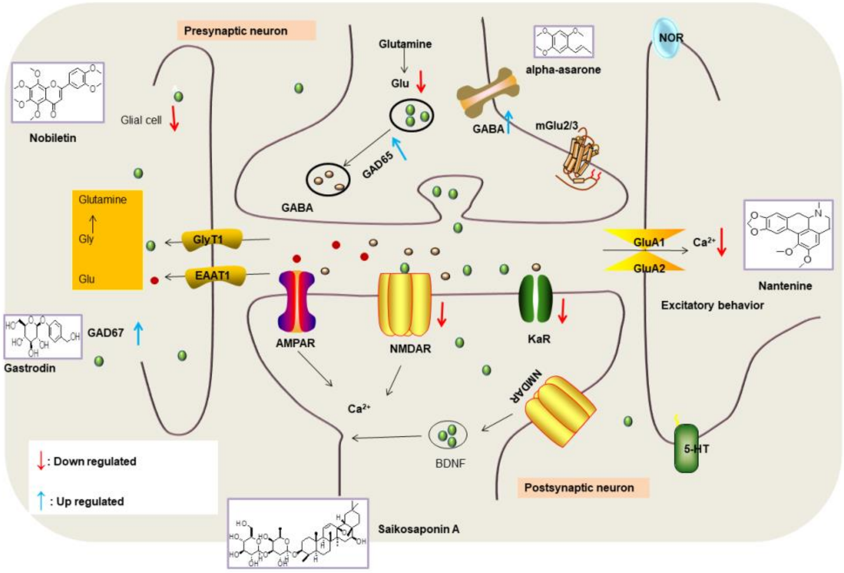


**Figure 1.** Effects of natural drugs on receptor pathway. α-amino-3-hydroxy-5-methyl-4-isoxazolepropionic acid receptor (AMPAR), brain-derived neurotrophic factor (BDNF), excitatory amino acid transporter (EAAT), γ-aminobutyric acid (GABA), glutamate decarboxylase (GAD), glutamate (Glu), glycine (Gly), N-methyl-D-aspartate receptor (NMDAR)

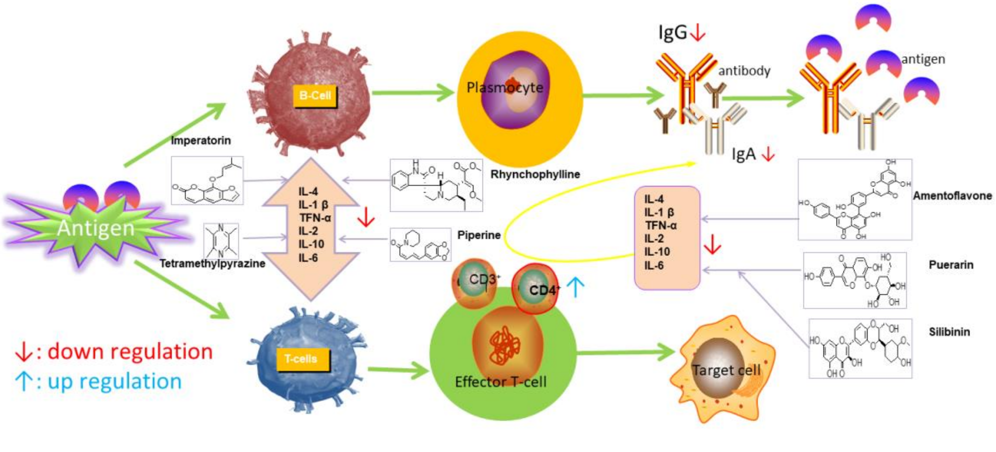


**Figure 2.** Effects of natural drugs on immune system. Interleukin 1β (IL-1β), interleukin 6 (IL-6), tumor necrosis factor-α (TNF-α)

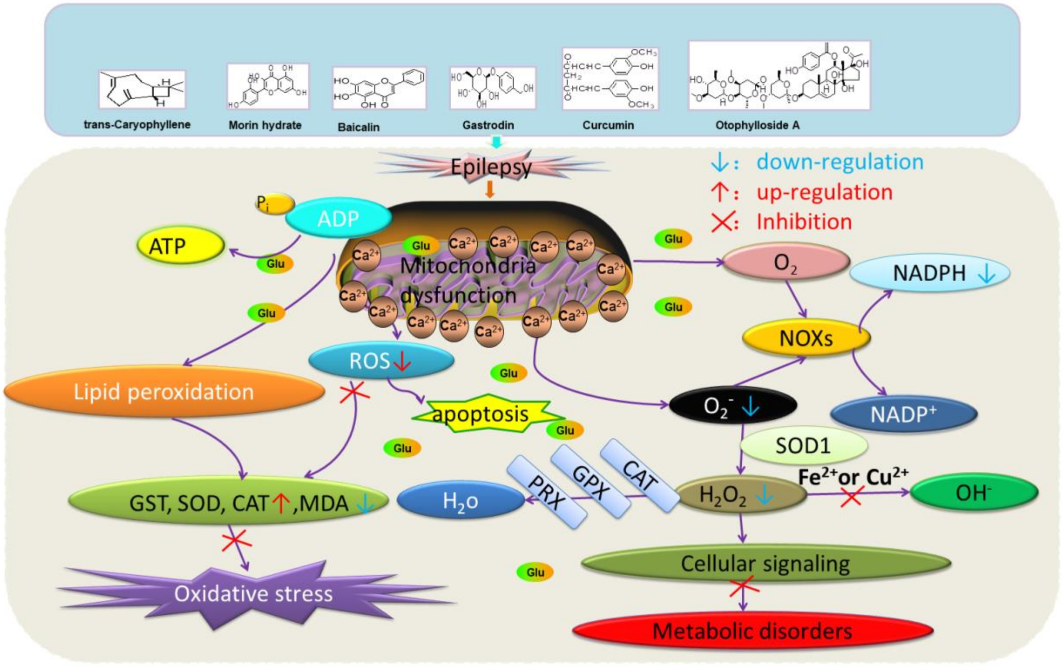


**Figure 3**. Effects of natural drugs on mitochondrial damage and oxidative stress. Adenosine triphosphate (ATP), adenosine diphosphate (ADP), catalase (CAT), glutathione S-transferase (GST), glutathione peroxidase (GPX), peroxiredoxin (PRX), malondialdehyde (MDA), reactive oxygen species (ROS), superoxide dismutase (SOD), nicotinamide adenine dinucleotide phosphate (NADPH) oxidase (NOXs)

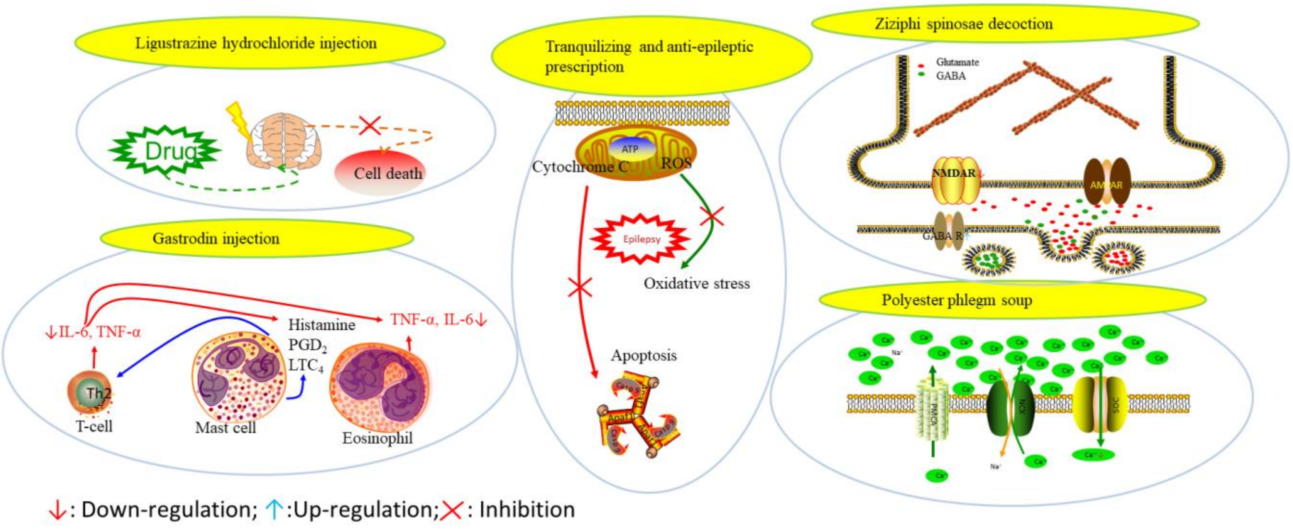


**Figure 4.** The therapeutic mechanism of integrated Chinese and Western medicine. Interleukin 6 (IL-6), reactive oxygen species (ROS), tumor necrosis factor-α (TNF-α), prostaglandin D2 ($PGD_2$), leukotriene C4 ($LTC_4$), γ-aminobutyric acid (GABA)
